# Blood levels of circulating methionine components in Alzheimer’s disease and mild cognitive impairment: A systematic review and meta-analysis

**DOI:** 10.3389/fnagi.2022.934070

**Published:** 2022-07-22

**Authors:** Yan Zhao, Xinyi Dong, Bingyu Chen, Yizhou Zhang, Sijia Meng, Fangzhen Guo, Xiaojing Guo, Jialei Zhu, Haoyue Wang, Huixian Cui, Sha Li

**Affiliations:** ^1^Department of Anatomy, Hebei Medical University, Shijiazhuang, China; ^2^School of Nursing, Hebei Medical University, Shijiazhuang, China; ^3^Neuroscience Research Center, Hebei Medical University, Shijiazhuang, China; ^4^Hebei Key Laboratory of Neurodegenerative Disease Mechanism, Shijiazhuang, China; ^5^The Key Laboratory of Neural and Vascular Biology of Ministry of Education, Hebei Medical University, Shijiazhuang, China

**Keywords:** methionine circulating, homocysteine, vitamin B_12_, blood biomarkers, Alzheimer’s disease, mild cognitive impairment

## Abstract

**Background:**

Circulating methionine components have been reported to be associated with Alzheimer’s disease (AD) and mild cognitive impairment (MCI), although outcomes are not always consistent.

**Materials and methods:**

Database searching was conducted using PubMed, Embase, Cochrane Library, and Web of Science from inception to 26 December 2021. In this study, two reviewers independently identified eligible articles and extracted the data. We used Joanna Briggs Institute (JBI) Critical Appraisal tools to assess the overall quality of the included studies. STATA software was employed to perform meta-analysis evaluating the standardized mean difference (SMD) with its 95% confidence intervals (CIs) using random-effects models. Evidence quality was assessed using the Grading of Recommendations Assessment, Development, and Evaluation (GRADE) criteria.

**Results:**

Totally, 30 observational studies were eligible for inclusion. Compared with cognitively normal controls, patients with AD had increased homocysteine (Hcy) levels in the blood [standardized mean difference (SMD) = 0.59, 95% confidence interval [CI]: 0.36–0.82, *P* = 0.000], plasma (SMD = 0.39, 95% CI: 0.23–0.55, *P* = 0.000), and serum (SMD = 1.56, 95% CI: 0.59–2.95, *P* = 0.002). Patients with MCI were not significantly different from controls (SMD = 0.26, 95% CI: –0.07–0.58, *P* = 0.127). Patients with AD or MCI did not significantly differ from controls of blood vitamin B_12_ levels, AD (SMD = –0.05, 95% CI: –0.19–0.08, *P* = 0.440), or MCI (SMD = 0.01, 95% CI: –0.16–0.17, *P* = 0.94). Some cohort studies have suggested that higher Hcy, methionine, and *S*-adenosylmethionine levels may accelerate cognitive decline in patients with MCI or AD, and vitamin B_12_ deficiency is a risk factor for the disease; however, the results of other studies were inconsistent. According to the GRADE system, all these outcomes scored very low to low quality, and no high-quality evidence was found.

**Conclusion:**

Only Hcy levels in the plasma and serum were found to be inversely related to the risk of AD. However, due to the low quality of supporting these results, high-quality studies are needed to verify these findings.

**Systematic Review Registration:**

http://www.crd.york.ac.uk/PROSPERO/, identifier CRD42022308961.

## Introduction

Alzheimer’s disease (AD) is a chronic neurodegenerative disease that is characterized by progressive memory loss and cognitive deficits. Globally, the incidence of AD doubles every 5 years after the age of 65 years. Furthermore, the number of cases is expected to exceed 115 million by 2050, placing a huge burden on patients, caregivers, and the society (Dubois et al., 2014; Mecca and van Dyck, 2021). Mild cognitive impairment (MCI) is a prodromal form of AD that is characterized by neurocognitive dysfunction (Chandra et al., 2019). However, pathological changes associated with AD begin to occur in the brain at least 10 years before the onset of overt symptoms and clinical manifestations, making the discovery of early diagnostic methods and timely interventions important for AD management (Petersen, 2009). This has led to intensive research on the discovery and development of accurate, reliable, and cost-effective early AD biomarkers (DeMarshall et al., 2016). Compared to other substances such as cerebrospinal fluid (CSF), blood is more readily available and can be used as a biomarker for AD screening and diagnosis. At the same time, blood is reproducible, non-invasive, simple, and economical and has a high level of sensitivity and specificity for AD (James et al., 2016). It can identify patients at risk for AD, progression from MCI to AD, and clinically confirmed rapid progression of AD (Altuna-Azkargorta and Mendioroz-Iriarte, 2018). Blood biomarkers would be a good approach for potential application as a screening tool in primary care or for longitudinal assessments of repeat sampling (Ritchie et al., 2017). An increasing number of patients with AD and lack of therapeutic drugs have prompted researchers in different fields to study identifiable and predictive risk factors to prevent the occurrence and development of AD. Among the recognizable substances, circulating methionine (Met) components are the most representative. In recent years, studies have reported an association between the components of the Met cycle in the blood and AD or MCI (Troesch et al., 2016; Singh and Prakash, 2017; Sklirou and Lichter-Konecki, 2018).

The components of the Met cycle include Met, *S*-adenosylmethionine (SAM), S-adenosylhomocysteine (SAH), and homocysteine (Hcy), wherein vitamin B_12_ is a coenzyme. DNA methylation and other epigenetic factors are important in the pathogenesis of AD. The metabolism of sulfur-containing amino acids in the transsulfuration pathway involves transfer of the sulfur atom of Met to serine to produce cysteine (Román et al., 2019). Met first reacts with ATP to form SAM, SAH, and Hcy. Remethylation defects can occur if Hcy fails to convert to the amino acid Met. This pathway requires the integrity of the gene encoding Methylenetetrahydrofolate reductase (MTHFR), which is required for the interaction between vitamin B_12_ and folate. Folate provides the methyl group required for the remethylation pathway to produce SAM, the main methyl donor for epigenetic processes (Román et al., 2019). MTHFR catalyzes the conversion of 5,10-methylenetetrahydrofolate to 5-methyltetrahydrofolate, a co-substrate of vitamin B_12_ for the remethylation of Hcy to Met (Román, 2015). It has been shown that elevated or decreased circulating Met components are strongly associated with the development of AD. A study by Pi et al. (2021) indicated that excessive or insufficient Met can lead to problems such as neuronal dysfunction and neurodegeneration, leading to symptoms such as AD. Elevated Hcy levels have been shown to be a risk factor for atrophy of specific brain regions and to be associated with cognitive decline (Singh and Prakash, 2017). Smith et al. (2018) and Wang et al. (2021) also concluded that AD is associated with higher Hcy levels. SAM is a direct metabolite of Met and is a methyl donor for almost all methylation reactions *in vivo* (Shrubsole et al., 2015), including DNA and RNA methylation (Chiang et al., 1996). Panza et al. (2009) suggested that SAM may be used as a neuroprotective dietary supplement in patients with AD. By contrast, some studies have shown the opposite. For example, Ravaglia et al. (2000a) and Miller et al. (2002) indicated that whether elevated Hcy directly influences AD pathogenesis or progression remains to be determined. Similarly, although there is a correlation between patients with AD with SAM and SAH (Kennedy et al., 2004; Li et al., 2017), the relationship between these groups needs to be evaluated in further studies (Sharma et al., 2017). Since vitamin B_12_ affects the Met cycle by acting as a cofactor for Met synthase (Lam et al., 2021), blood levels of vitamin B_12_ may also affect the levels of Met cycle components, with consequent effects on the development of AD or MCI. Some studies have found a significant association between vitamin B_12_ concentrations and cognitive decline (Tangney et al., 2009; Yu et al., 2016), but others have not (O’Leary et al., 2012; Zhang et al., 2017). The association between vitamin B_12_ and Hcy levels and cognitive decline suggests a significant role in AD/MCI. However, studies have provided conflicting and inconsistent results regarding the association between circulating blood Met components and cognitive decline, and the available evidence does not adequately indicate a link between the two factors. To the best of our knowledge, no formal meta-analysis has been conducted on the blood levels of circulating Met components in AD or MCI patients.

The aim of this systematic review and meta-analysis was to determine the relationship between circulating Met components and AD/MCI.

## Materials and methods

This meta-analysis was registered in PROSPERO (CRD42022308961) and conducted according to the Preferred Reporting Items for Systematic Reviews and Meta-Analyses guidelines (Page et al., 2021).

### Search strategy

We searched for potentially eligible studies using PubMed, Embase, Cochrane Library, and Web of Science databases from inception to December 26, 2021. Searches of all English databases included the following terms: (“Alzheimer’s disease” OR “Alzheimer*” OR “cognitive impairment”) AND (“methionine” OR “Met” OR “*S*-adenosylmethionine” OR “*S*-adenosyl methionine” OR “*S*-adenosyl-*L*-methionine” OR “SAM” OR “SAMe” OR “AdoMet” OR “*S*-adenosyl homocysteine” OR “*S*-adenosyl-*L*-homocysteine” OR “SAH” OR “AdoHcy” OR “amino acid*” OR “methylation” OR “transmethylation” OR “methyl group” OR “methyl donor”) AND (“biomarker*” OR “RBC*” OR “serum” et al.). All search terms are listed in the Supplementary Material. Database searches were supplemented by expert contact, reference and citation checking, and gray literature.

### Inclusion criteria

Studies were included if they simultaneously met the following criteria: (a) studies that provided the diagnostic criteria for AD or MCI used; (b) the study types were case–control, cohort, or cross-sectional; and (c) human studies that reported the peripheral blood levels of any of the most representative biomarkers (e.g., Hcy, Met, SAM, SAH, and the SAM/SAH ratio) of circulating Met components in patients with AD or MCI and healthy controls.

### Exclusion criteria

Studies were excluded if they (a) were duplicate publications; (b) were reviews, meta-analyses, case reports, letters, non-human studies, conference abstracts, or did not present the original data; (c) were animal model studies; (d) were studies without baseline data; or (e) were missing necessary data (e.g., SAM and SAH) or healthy controls or included fewer than 10 people in the case or control groups. We also excluded (f) studies reporting on drugs or measures affecting blood Hcy, Met, SAM, and SAH levels unless they provided basal blood Hcy, Met, SAM, and SAH level data prior and after the intervention.

### Data extraction

The following data have been extracted independently and cross-checked by two authors: author, year of publication, country, sample size, age, sex (% of male), matching factors, diagnosis, diagnostic method, sample source and units of measure, biomarkers, and analytical technology. The variables extracted from each are listed in Table 1. Finally, 15 eligible articles were included in the meta-analysis; 15 cross-sectional or cohort studies were included in the qualitative synthesis due to the heterogeneity or lack of necessary data. Standard error (SE) was converted into standard deviation (SD) by applying the following computational method: SD equals the SE multiplied by the square root of the sample size. If data were reported as medians and interquartile ranges (IQRs), they were transformed into means and SD using the methodology of Wan et al. (2014).

**TABLE 1:** Characteristics of the included studies.

Study	Study design	Region	Sample size			AGE (mean ± SD)			Gender (m/f)			Sample source			Risk of bias	Included in meta-analysis?
			AD	Control	MCI	AD	Control	MCI	AD	Control	MCI	Plasma	Serum	Blood		
Grossi et al. (2016)	Case-control	Italy	100	100	-	77.5 ± 7.5	76.8 ± 8.4	-	46/54	46/54	-	Homocysteine	Vitamin B_12_	no	LOW	yes
Miller et al. (2002)	Case-control	California	43	37	-	78.0 ± 7.0	75.0 ± 7.0	-	13/19	8/14	-	yes	no	no	LOW	yes
Veryard et al. (2013)	Case-control	United Kingdom	40	30	-	82.4 ± 1.0	78.0 ± 1.6	-	23/17	10/20	-	yes	no	no	LOW	no
Selley (2004)	Case-control	Australia	25	25	-	75.6 ± 6.2	74.8 ± 4.7	-	12/13	12/13	-	yes	no	no	LOW	yes
Piazza et al. (2012)	Case-control	Italy	110	100	38	73.9 ± 5.0	71.9 ± 5.4	72.5 ± 5.0	58/52	43/57	11/27	Homocysteine	Vitamin B_12_	no	LOW	yes
Tannorella et al. (2015)	Case-control	Italy	120	115	-	76.9 ± 8.1	76.3 ± 8.3	-	49/71	51/64	-	Homocysteine	Vitamin B_12_	no	LOW	yes
Cervellati et al. (2014)	Case-control	Italy	143	48	103	79.0 ± 6.4	77.8 ± 4.9	77.8 ± 4.1	47/96	15/33	37/66	yes	no	no	LOW	yes
Clarke et al. (1998)	Case-control	England	240	108		74.3 ± 8.5	72.8 ± 8.8	-	148/92	62/46	-	no	yes	no	LOW	yes
Bednarska-Makaruk et al. (2016)	Case-control	Poland	53	45	47	70.9 ± 8.4	71.8 ± 6.5	69.0 ± 8.2	11/42	20/25	23/24	homocysteine	vitamin B_12_	no	LOW	yes
McCaddon et al. (1998)	Case-control	Wrexham	30	30	-	79.0 ± 1.8	79.0 ± 2.5	-	10/20	10/20	-	no	yes	no	LOW	yes
Hooshmand et al. (2019)	Cohort	Sweden	241	2329	-	82.8 ± 7.6	72.2 ± 10.1	-	63/178	929/1440	-	no	no	yes	LOW	no
Blasko et al. (2008)	Cohort	Austria	98	389	-	75.8 ± 0.5		-	-			yes	no	no	LOW	no
de Leeuw et al. (2020a)	Cohort	Amsterdam	100	36	45	66.8 ± 8.4	63.7 ± 8.1	66.9 ± 8.2	50/50	195/166	26/19	yes	yes	no	LOW	no
de Leeuw et al. (2020b)	Cohort	Amsterdam	29	220	100	65.1 ± 8.3	62.1 ± 9.7	67.8 ± 7.2	18/11	132/88	66/34	yes	no	no	LOW	no
Annerbo et al. (2009)	Coort	Stockholm	61	139	-	82.7 ± 4.6	80.1 ± 4.3	-	19/64	35/95	-	yes	no	no	LOW	no
Seshadri et al. (2002)	Cohort	Framingham	Men: 425		–	Men: 76.0 ± 5.0		–	425/667			Yes	No	No	LOW	No
			Women: 667			Women: 77.0 ± 6.0										
Annerbo et al. (2005)	Cohort	Sweden	30	66	-	67.8 ± 7.5	62.5 ± 11.0	-	18/12	32/34	-	yes	no	no	LOW	no
Annerbo et al. (2006)	Cohort	Sweden	31	61	-	67.7 ± 7.2	63.6 ± 9.6	-	32/29	13/19	-	no	no	yes	LOW	no
McCaddon et al. (2001)	Cohort	North Wales	Men: *n* = 10		–	79.0 ± 2.5		–	10/22		–	No	No	Yes	LOW	No
			Women: *n* = 22													
Budge et al. (2000)	Cohort	United Kingdom	156		-	74.1 ± 6.2		-	76/80		-	no	no	yes	LOW	no
Cankurtaran et al. (2013)	Cross sectional	Turkey	143	1553	-	73.6 ± 6.3	72.5 ± 5.9	-	92/51	965/588	-	yea	no	no	LOW	yes
Frick et al. (2006)	Cross sectional	Austria	30	-	16	78.6 ± 11.0	-	76.6 ± 6.0	7/23	-	8/8	no	yes	no	MODERATE	no
Raszewski et al. (2016)	Cross sectional	Lublin Region	64	40	-	74.3 ± 7.6	73.6 ± 9.4	-	22/42		-	no	yes	no	MODERATE	yes
Faux et al. (2011)	Cross sectional	Australia	205	760	130	78.4 ± 8.7	70 ± 7	75.7 ± 7.6	80/125	323/437	56/74	Homocysteine	vitamin B_12_	no	LOW	yes
Dayon et al. (2017)	Cross sectional	Switzerland	48	72	-	66.0 ± 7.4	73.3 ± 6.9	-	17/31	26/46	-	yes	no	no	LOW	yes
Stewart et al. (2002)	Cross sectional	African-Caribbean	238			65.1 ± 5.4			111/127			yes	no	no	LOW	no
Corso et al. (2017)	Cross sectiona	Italy	29	46	18	71.4 ± 9.5	65.0 ± 7.1	69.4 ± 8.2	7/22	20/26	4/14	no	yes	no	LOW	no
Devčić et al. (2014)	Cross sectiona	Croatia	55	-	-	80.8 ± 6.4	-	-	Female	-	-	no	yes	no	LOW	no
Lehmann et al. (1999)	Cross sectional	Sweden	172	69	-	72.7 ± 7.7	64.9 ± 8.5	-	151/185		-	Vitamin B_12_	Homocysteie	no	LOW	yes
Linnebank et al. (2010)	Cross sectional	Switzerland	60	60	-	73.0 ± 8.0	62 ± 10	-	17/43	62 ± 10	-	no	no	yes	LOW	yes

AD, Alzheimer’s disease; MCI, mild cognitive impairment; -, data not available.

### Quality evaluation

Joanna Briggs Institute (JBI) Critical Appraisal tools^1^ were used to assess the study quality and the risk of bias in eligible studies (Moola et al., 2019). Each tool composed of 8 to 11 domains; “yes,”, “no,” “unclear,” and “not applicable” were used to answer, respectively, depending on the study design (detailed description see Supplementary File 1). According to the guidelines (Munn et al., 2020), thresholds are best decided by the two systematic reviewers. The risk of bias of the studies was categorized as low risk of bias (70% or more ‘‘yes’’ responses), moderate risk of bias (50--69% ‘‘yes’’ responses), and high risk of bias (up to 49% ‘‘yes’’ responses). Overall, we considered the methodological quality of studies incorporated into our meta-analysis. The GRADE methodology was used to evaluate the quality of the body of retrieved evidence (GRADEpro).^2^ The GRADE system assesses evidence quality with four levels: high, moderate, low, or very low. The initial grading would be decreased if there were study limitations, inconsistencies, imprecision, indirectness, or publication bias (Atkins et al., 2004).

### Statistical analysis

Meta-analyses were performed using STATA (version 16.0, StataCorp, College Station, TX, United States) with random-effects models. First, a Q-test was used to verify whether there was heterogeneity among the included models. The degree of heterogeneity was assessed using the I^2^ statistic, with I^2^ values of 25, 50, and 75% being considered to indicate low, moderate, and high heterogeneity, respectively. If I^2^ > 50%, heterogeneity was considered larger, and the random-effects model was used for analysis; otherwise, the fixed effects model was used. The count data were represented by standardized mean differences (SMDs) and 95% CI. Sensitivity analyses were conducted by successively removing one study at a time to confirm whether any single study affected the meta-analysis results (leave-one-out analysis). Subgroup analyses were performed based on the sample source, and each subgroup included at least three studies. Publication bias was assessed by visual inspection of funnel plots to aid judgement.

## Results

### Literature search

**FIGURE 1:**
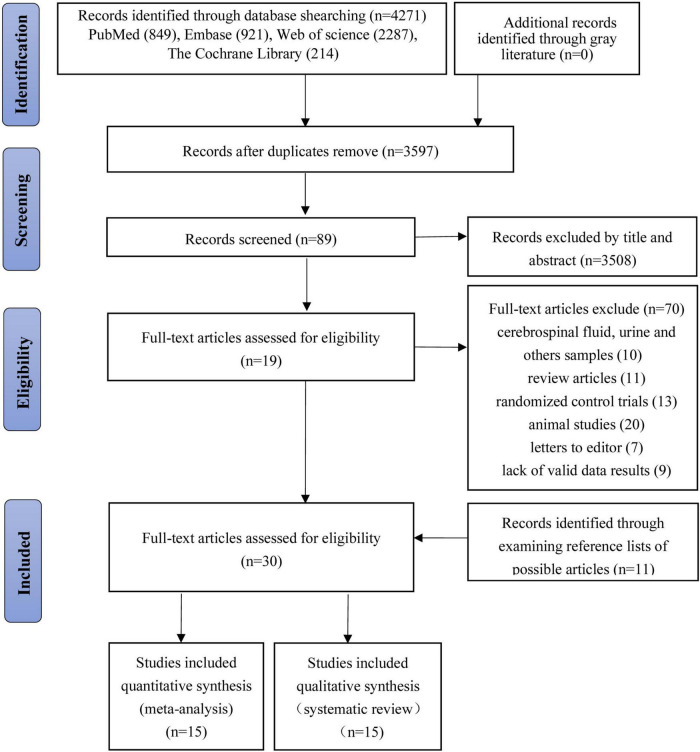
Flowchart for searching and selection of the included studies.

We retrieved 4,271 studies based on the retrieval strategy (PubMed = 849, Embase = 921, Web of Science = 2,287, The Cochrane Library = 214). Ultimately, we included 10 case–control studies, 10 cohort studies, and 10 cross-sectional studies, that is, 30 studies in total (Clarke et al., 1998; McCaddon et al., 1998, 2001; Lehmann et al., 1999; Budge et al., 2000; Miller et al., 2002; Seshadri et al., 2002; Stewart et al., 2002; Selley, 2004; Annerbo et al., 2005, 2006, 2009; Frick et al., 2006; Blasko et al., 2008; Linnebank et al., 2010; Faux et al., 2011; Piazza et al., 2012; Cankurtaran et al., 2013; Veryard et al., 2013; Cervellati et al., 2014; Devčić et al., 2014; Tannorella et al., 2015; Bednarska-Makaruk et al., 2016; Grossi et al., 2016; Raszewski et al., 2016; Corso et al., 2017; Dayon et al., 2017; Hooshmand et al., 2019; de Leeuw et al., 2020a,b; Figure 1).

### Study characteristics

Most articles were published between 2000 and 2020, two studies were published in 1998, and only one study was published in 1999. A total of 23 studies were conducted in Europe (Clarke et al., 1998; McCaddon et al., 1998, 2001; Lehmann et al., 1999; Budge et al., 2000; Annerbo et al., 2005, 2006, 2009; Frick et al., 2006; Linnebank et al., 2010; Piazza et al., 2012; Veryard et al., 2013; Cervellati et al., 2014; Devčić et al., 2014; Tannorella et al., 2015; Bednarska-Makaruk et al., 2016; Grossi et al., 2016; Raszewski et al., 2016; Corso et al., 2017; Dayon et al., 2017; Hooshmand et al., 2019; de Leeuw et al., 2020a,b), two studies in North America (Miller et al., 2002; Seshadri et al., 2002), three studies in Oceania (Selley, 2004; Blasko et al., 2008; Faux et al., 2011), one study in Africa (Stewart et al., 2002), and one study in Asia (Cankurtaran et al., 2013).

Homocysteine levels in patients with AD were studied in all 15 articles (Clarke et al., 1998; Lehmann et al., 1999; Miller et al., 2002; Selley, 2004; Linnebank et al., 2010; Faux et al., 2011; Piazza et al., 2012; Cankurtaran et al., 2013; Veryard et al., 2013; Cervellati et al., 2014; Tannorella et al., 2015; Bednarska-Makaruk et al., 2016; Grossi et al., 2016; Raszewski et al., 2016; Dayon et al., 2017) that were included in the quantitative analysis, and a total of 4,788 people participated (1,618 cases and 3,170 controls). Overall, three of these articles (Faux et al., 2011; Piazza et al., 2012; Cervellati et al., 2014) researched serum/plasma levels of Hcy in patients with MCI, including 1,179 people (217 cases and 908 controls); 11 articles (Clarke et al., 1998; Lehmann et al., 1999; Miller et al., 2002; Linnebank et al., 2010; Piazza et al., 2012; Cankurtaran et al., 2013; Tannorella et al., 2015; Bednarska-Makaruk et al., 2016; Grossi et al., 2016; Raszewski et al., 2016; Dayon et al., 2017) reported the serum/plasma levels of vitamin B_12_ in patients with AD, including 4,367 people (1,348 cases and 3,019 control); two articles (Faux et al., 2011; Piazza et al., 2012) reported serum or plasma levels of vitamin B_12_ with MCI, including 1,208 people (168 cases and 860 controls).

A total of 15 articles (McCaddon et al., 1998, 2001; Budge et al., 2000; Seshadri et al., 2002; Stewart et al., 2002; Annerbo et al., 2005, 2006, 2009; Frick et al., 2006; Blasko et al., 2008; Devčić et al., 2014; Corso et al., 2017; Hooshmand et al., 2019; de Leeuw et al., 2020a,b) were included in the qualitative studies due to a lack of necessary data; 14 articles (Budge et al., 2000; McCaddon et al., 2001; Seshadri et al., 2002; Stewart et al., 2002; Annerbo et al., 2005; Annerbo et al., 2006; Frick et al., 2006; Blasko et al., 2008; Annerbo et al., 2009; Veryard et al., 2013; Devčić et al., 2014; Hooshmand et al., 2019; de Leeuw et al., 2020a,b) reported on Hcy levels in the patients’ blood, 10 articles (Budge et al., 2000; McCaddon et al., 2001; Seshadri et al., 2002; Annerbo et al., 2005, 2006; Frick et al., 2006; Blasko et al., 2008; Hooshmand et al., 2019; de Leeuw et al., 2020a,b) reported on blood levels of vitamin B_12_, two studies (Corso et al., 2017; Hooshmand et al., 2019) reported on levels of Met, one article (de Leeuw et al., 2020b) reported on SAM levels, and one article reported on SAH levels in the blood.

### Results of quality and risk of bias

The bias risk assessment showed that among the included articles, 28 respected all the JBI criteria for a good-quality study (low risk), and two articles showed moderate risk (Supplementary File 2).

### Results of the meta-analysis: Homocysteine levels

#### Alzheimer’s disease vs. controls

**FIGURE 2:**
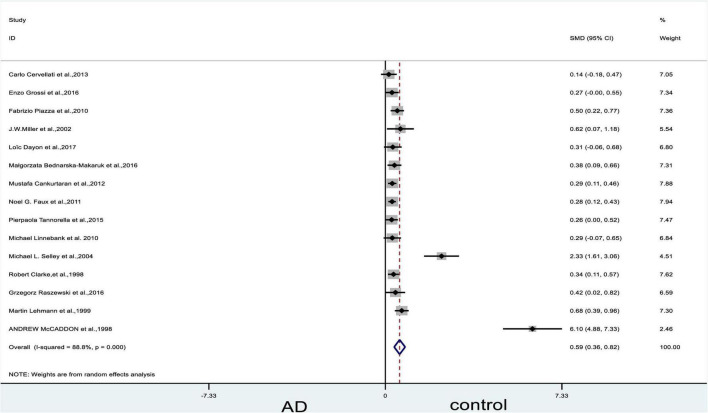
Forest plot of 15 studies comparing blood Hcy levels between AD and control. SMD, standardized mean difference; CI, confidence interval; Hcy, homocysteine; AD, Alzheimer’s disease; Control, cognitive normal.

The meta-analysis of 15 studies (4,788 participants) assessing Hcy levels in blood showed moderate increases in Hcy levels in patients with AD compared to controls (SMD = 0.59, 95% confidence interval [CI]: 0.36–0.82, *P* = 0.000); the difference was statistically significant. Significant heterogeneity was observed among the studies (X^2^ = 120.73, I^2^ = 88.8%, *P* = 0.000) (Figure 2). According to the GRADE system, the overall level of evidence with respect to Hcy on AD and controls was “very low” (Table 2).

**TABLE 2:** GRADE quality of evidence assessment of outcome indicators for the included studies.

Outcome indicator	Number of included cases	Heterogeneity		Model of analysis	Group effect value		Estimated value	95% CI	GRADE
		I^2^	*p*		*Z*	*p*			
Hcy levels between AD vs. Control	4,788	88.8%	<0.0001	Random effect	5.08	<0.000	0.59 (SMD)	0.36, 0.28	Very low
Vitamin B_12_ levels between AD vs. Control	4,367	67.5%	0.001	Random effect	0.77	0.440	–0.05 (SMD)	–0.19, 0.08	Low
Vitamin B_12_ levels between MCI vs. Control	1,028	0.0%	0.56	Random effect	0.07	0.941	0.01 (SMD)	–0.16, 0.17	Very low
Hcy levels between MCI vs. Control	1,179	72.5%	0.026	Random effect	1.53	0.127	0.26 (SMD)	–0.07, 0.58	Low

SMD, standardized mean difference; CI, confidence interval; GRADE, grading of recommendation assessment, development, and evaluation.

#### Subgroup analysis on homocysteine levels: Categorized by sample source

**FIGURE 3:**
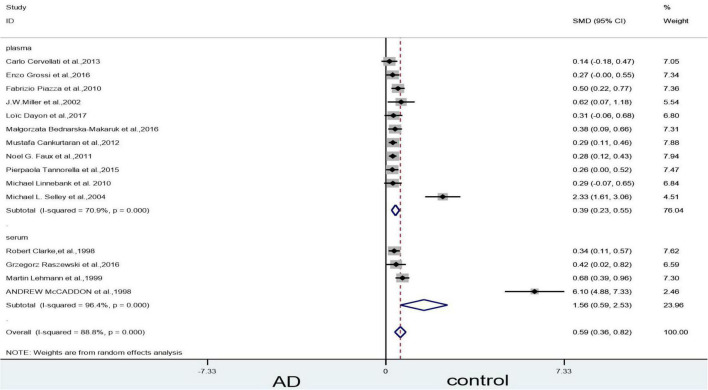
Forest plot of 15 studies comparing blood Hcy levels between AD and control: sub-grouped by sample source. SMD, standardized mean difference; CI, confidence interval; Hcy, homocysteine; AD, Alzheimer’s disease; Control, cognitive normal.

The subgroup analysis revealed that Hcy levels were higher in the diseased group than in the control group, regardless of whether the sample source was the serum or the plasma (plasma: SMD = 0.39, 95% CI: 0.23–0.55, *P* = 0.000; serum: SMD = 1.56, 95% CI: 0.59-2.95, *P* = 0.002). Heterogeneity tests on plasma (X^2^ = 34.34, I^2^ = 70.9%, *P* = 0.000) and serum (X^2^ = 84.00, I^2^ = 96.4%, *P* = 0.000) yielded highly significant results (Figure 3).

#### Sensitivity analyses

**FIGURE 4:**
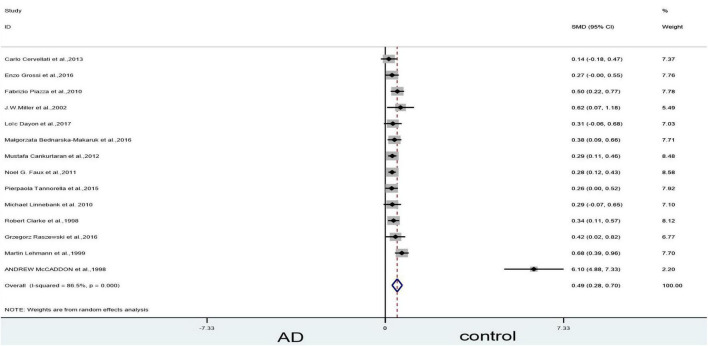
Sensitivity meta-analysis of 14 studies comparing blood Hcy levels between AD and control by removing the article of Selley (2004). SMD, standardized mean difference; CI, confidence interval; Hcy, homocysteine; AD, Alzheimer’s disease; Control, cognitive normal.

**FIGURE 5:**
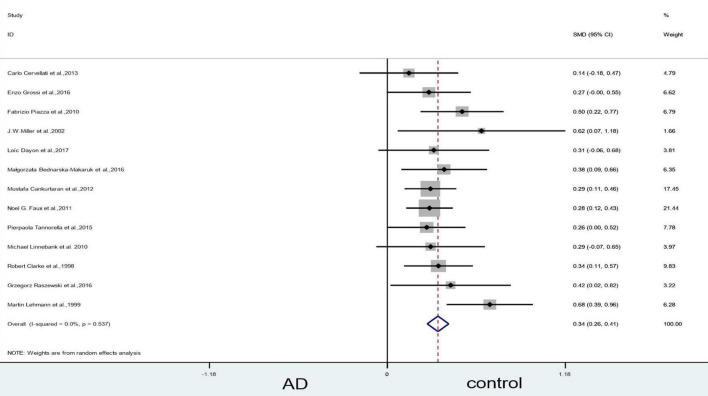
Sensitivity meta-analysis of 13 studies comparing blood Hcy levels between AD and control by removing the article of Selley (2004) and McCaddon et al. (1998). SMD, standardized mean difference; CI, confidence interval; Hcy, homocysteine; AD, Alzheimer’s disease; Control, cognitive normal.

**FIGURE 6:**
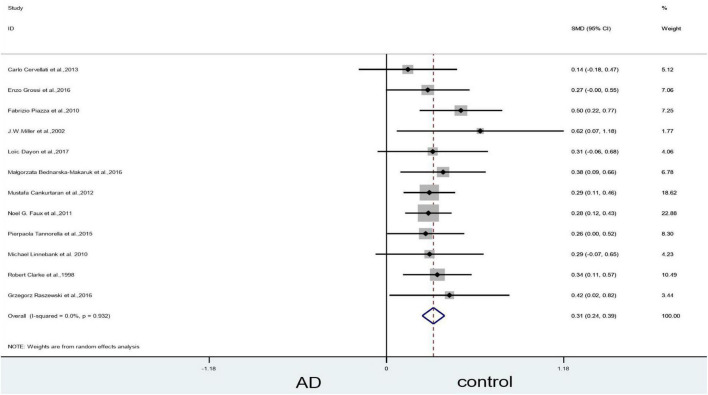
Sensitivity meta-analysis of 12 studies comparing blood Hcy levels between AD and control by removing the article of Selley (2004), McCaddon et al. (1998) and Lehmann et al. (1999). SMD, standardized mean difference; CI, confidence interval; Hcy, homocysteine; AD, Alzheimer’s disease; Control, cognitive normal.

Sensitivity analyses suggested that three articles (McCaddon et al., 1998; Lehmann et al., 1999; Selley, 2004) could influence the statistically significant difference in the blood levels of Hcy. When we removed Selley (2004) article, the heterogeneity (X^2^ = 96.40, I^2^ = 86.5%, *P* = 0.000), and combined effect size decreased slightly (SMD = 0.49, 95% CI: 0.28–0.70, *P* = 0.000); however, the difference remained statistically significant (Figure 4). When we excluded the article of McCaddon et al. (1998) article, which yielded non-significant results (X^2^ = 10.91, I^2^ = 0, *P* = 0.537) (Figure 5). Finally, when we removed the aforementioned two articles, with Lehmann et al. (1999), article heterogeneity was completely absent (X^2^ = 4.99, I^2^ = 0, *P* = 0.932), and the combined effect size (SMD = 0.31, 95% CI: 0.24–0.39, *P* = 0.000) (Figure 6) was statistically significant. The two researchers came to the conclusion that the aforementioned three articles add heterogeneity in terms of methodology and participants.

#### Publication bias

The funnel plot test results showed that two articles fall outside the dashed line, and one article intersects with the dashed line; heterogeneity in the other articles appeared roughly symmetric. This finding suggested that there was no significant publication bias (Supplementary Figure 1).

#### Mild cognitive impairment vs. controls

**FIGURE 7:**
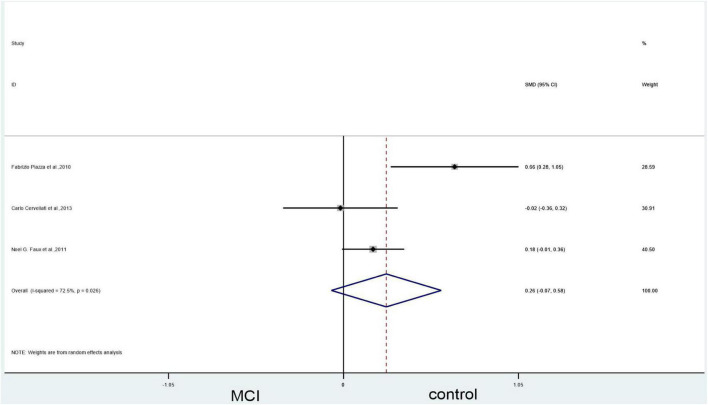
Forest plot of three studies comparing blood Hcy levels between MCI and control. SMD, standardized mean difference; CI, confidence interval; Hcy, homocysteine; MCI, mild cognitive impairment; Control, cognitive normal.

Forest plots showed no significant difference between blood Hcy levels in patients with MCI and those in controls (SMD = 0.26, 95% CI: –0.07–0.58, *P* = 0.127), but there was significant heterogeneity (X^2^ = 7.26, I^2^ = 72.5%, *P* = 0.026) (Figure 7). However, since we included only three articles, bias might have occurred. GRADE results display that the level of evidence with respect to the level of Hcy on MCI and controls was “low” (Table 2).

### Results of the meta-analysis: Vitamin B_12_ levels

#### Alzheimer’s disease vs. controls

**FIGURE 8:**
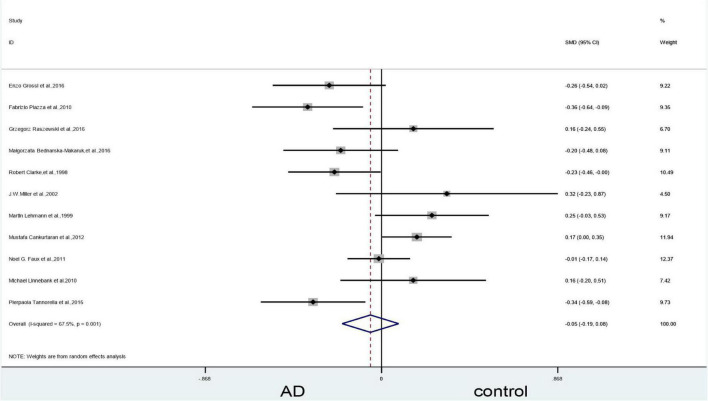
Forest plot of 11 studies comparing blood vitamin B_12_ levels between AD and control. SMD, standardized mean difference; CI, confidence interval; AD, Alzheimer’s disease; Control, cognitive normal.

An analysis of the 11 included articles found that there was no statistically significant difference in vitamin B_12_ levels in the blood between patients with AD and controls (SMD = –0.05, 95% CI: –0.19–0.08, *P* = 0.440); there was, however, significant heterogeneity (X^2^ = 30.73, I^2^ = 67.5%, *P* = 0.001) (Figure 8). The quality of evidence using GRADE’s summary of vitamin B_12_ levels between AD vs. controls was judged to be low (Table 2).

#### Subgroup analysis of vitamin B_12_: Categorized by sample source

**FIGURE 9:**
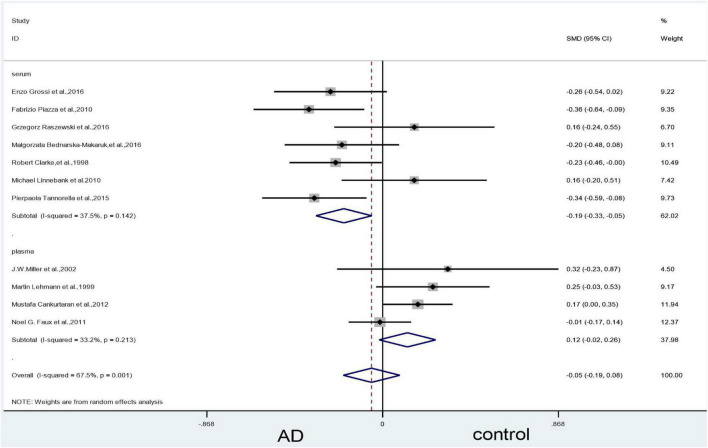
Forest plot of 11 studies comparing blood vitamin B_12_ levels between AD and control: sub-grouped by sample source. SMD, standardized mean difference; CI, confidence interval; AD, Alzheimer’s disease; Control, cognitive normal.

The subgroup analysis based on sample source of plasma showed no significant difference in vitamin B12 levels between AD patients and controls (SMD = 0.12, 95% CI: –0.02–0.26, *P* = 0.086), but the results in the serum showed differences (SMD = –0.19, 95% CI: –0.33–0.05, *P* = 0.006). There was, however, significant heterogeneity (Figure 9).

#### Sensitivity analyses

**FIGURE 10:**
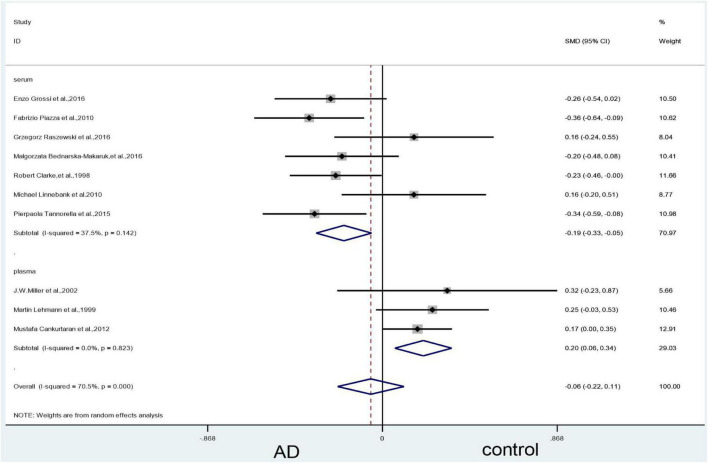
Sensitivity meta-analysis of 10 studies comparing blood vitamin B_12_ levels between AD and control by removing the article of Faux et al. (2011). SMD, standardized mean difference; CI, confidence interval; AD, Alzheimer’s disease; Control, cognitive normal.

In the plasma subgroup, we removed the study by Faux et al. (2011), which led to a significant decrease in heterogeneity (X^2^ = 0.39, I^2^ = 0%, *P* = 0.823). The combined effect sizes showed higher vitamin B_12_ levels in the control group than in the AD group, with statistically significant differences (SMD = 0.20, 95% CI: 0.06–0.34, *P* = 0.005) (Figure 10).

**FIGURE 11:**
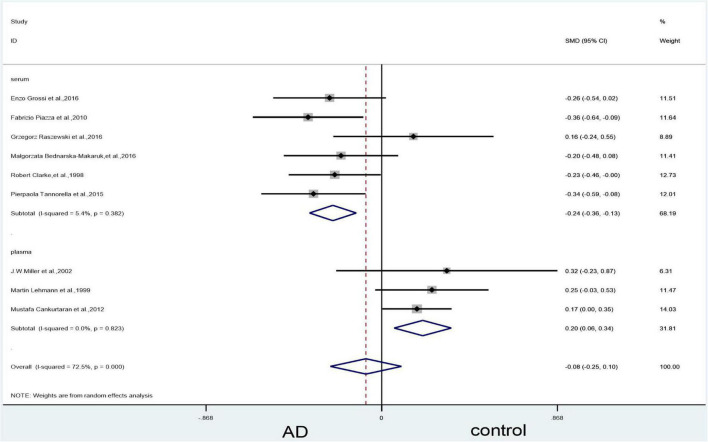
Sensitivity meta-analysis of 9 studies comparing blood vitamin B_12_ levels between AD and control by removing the article of Faux et al. (2011) and Linnebank et al. (2010). SMD, standardized mean difference; CI, confidence interval; AD, Alzheimer’s disease; Control, cognitive normal.

**FIGURE 12:**
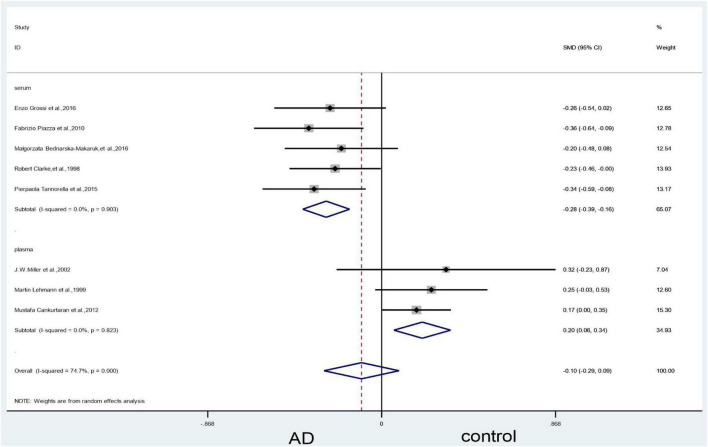
Sensitivity meta-analysis of 8 studies comparing blood vitamin B_12_ levels between AD and control by removing the article of Faux et al. (2011), Linnebank et al. (2010) and Raszewski et al. (2016). SMD, standardized mean difference; CI, confidence interval; AD, Alzheimer’s disease; Control, cognitive normal.

In the serum subgroup, when we removed Linnebank et al. (2010) article the heterogeneity test results changed to 5% (X^2^ = 5.28, I^2^ = 5.4%, *P* = 0.39); the difference was statistically significant (SMD = –0.24, 95% CI: –0.36—0.13, *P* = 0.000) (Figure 11). When we removed Raszewski et al. (2016); Linnebank et al. (2010) and Faux et al. (2011) articles simultaneously, the results of the meta-analysis did not significantly change (Figure 12).

#### Publication bias

A funnel plot test on vitamin B_12_ showed that four articles were located outside the dashed line, and publication bias was indicated (Supplementary Figure 2).

#### Mild cognitive impairment vs. controls

**FIGURE 13:**
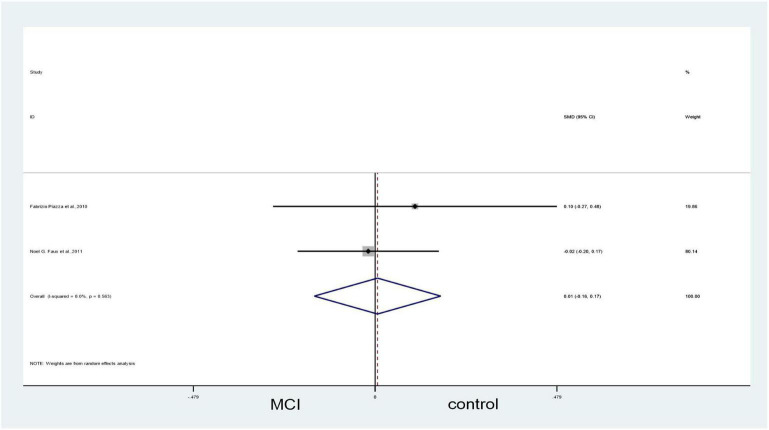
Forest plot of 2 studies comparing blood vitamin B_12_ levels between MCI and control. SMD, standardized mean difference; CI, confidence interval; MCI, mild cognitive impairment: Control, cognitive normal.

Forest plots showed that there was no statistically significant difference in vitamin B_12_ levels between the MCI and control groups (SMD = 0.01, 95% CI: –0.16–0.17, *P* = 0.94), as well as no heterogeneity (X^2^ = 0.33, I^2^ = 0%, *P* = 0.56) (Figure 13). The level of evidence is “very low” (Table 2).

### Results of evidence quality

Four outcomes were evaluated by using the GRADE system. According to the evaluation results, no high-quality evidence was found, two outcomes provided low evidence, and two provided “very low.” The details are given in Table 2.

### Result of the qualitative analysis

#### Homocysteine levels and the risk of Alzheimer’s disease or mild cognitive impairment

Totally, nine studies (Seshadri et al., 2002; Stewart et al., 2002; Annerbo et al., 2006, 2009; Frick et al., 2006; Devčić et al., 2014; Hooshmand et al., 2019; de Leeuw et al., 2020a,b) have reported an association between Hcy and AD/MCI, and high levels of Hcy have been associated with the risk of AD/MCI. Hcy levels were obviously higher in AD than in healthy individuals, and plasma Hcy levels were higher than stable levels during the clinical progression of MCI and AD. Furthermore, one study (Annerbo et al., 2005) reported that hyperhomocysteinemia is not positively associated with the development of AD but that low levels of Hcy can play a protective role in the process of MCI translation into AD. Conversely, in a report by Veryard et al. (2013), there was no marked difference in plasma Hcy levels between AD and control groups.

#### Vitamin B_12_ level with the risk of Alzheimer’s disease or mild cognitive impairment

Overall, three articles (Frick et al., 2006; Blasko et al., 2008; Annerbo et al., 2009) mentioned an association between vitamin B_12_ and patients’ cognitive conditions. Two of these studies suggested that low levels of vitamin B_12_ are associated with poor cognitive performance and an increased risk of mental disorders or AD (Frick et al., 2006; Annerbo et al., 2009). Vitamin B_12_ supplementation for 1 month significantly reduced Hcy levels and improved the cognitive status of patients. Blasko et al. (2008) indicated that changes in vitamin B_12_ levels were inversely correlated with Hcy. Different from the aforementioned studies, Stewart et al. (2002) and Annerbo et al. (2006) reported that there was no significant association between vitamin B_12_ and AD.

#### Methionine and *S*-adenosylmethionine levels with the risk of Alzheimer’s disease or mild cognitive impairment

In all, two studies (Corso et al., 2017; Hooshmand et al., 2019) examined the relationship between Met levels and AD, and one cohort study (Hooshmand et al., 2019) showed that elevated serum Met levels may be associated with brain atrophy and dementia risk in older adults. In the study by Corso et al. (2017), there was no specific association between Met and AD or MCI, but Met levels were found to affect the incidence of AD/MCI. In a cohort study (de Leeuw et al., 2020b), the investigators found that excessive SAM in the blood may be a risk factor for cognitive decline. Due to the limitation of the eligible literature, the qualitative analysis of Met and SAM reported here may have been biased.

## Discussion

This systematic review and meta-analysis, based on the available data of case–control, cohort, and cross-sectional studies, synthesizes current evidence on the association between blood Met cycle component levels and AD/MCI. In summary, our meta-analysis demonstrated five points: (a) Individuals with AD compared with controls had significantly increased levels of Hcy. (b) For people with MCI, there was no significant difference in blood Hcy levels in the control group. (c) No differences in blood vitamin B12 levels were found between patients with AD or MCI and controls. (d) Sensitivity analyses indicated that the main outcomes of the meta-analysis were affected by some studies, but the results remained stable after exclusion of these studies. (e) The results of our subgroup analyses were consistent with the overall results. Totally, two points for qualitative analysis were concluded: (a) A majority of the included cohort studies suggested that higher Hcy, Met, and SAM levels may accelerate cognitive decline in patients with MCI or AD, and (b) vitamin B_12_ deficiency may be a risk factor for the disease. Two points for dietary factor were arrived: (a) Faux et al. (2011), referring to dietary control, suggested that dietary supplementation with vitamin B_12_ or folic acid reduces plasma Hcy, with no corresponding cognitive improvement, despite successful Hcy reduction. (b) Totally, three articles (Seshadri et al., 2002; Piazza et al., 2012; Cankurtaran et al., 2013) recorded dietary habits, alcohol intake, and diabetic diet separately but found no effects on outcomes. It is worth mentioning that only few studies contained analyses of MCI and research of diet, which may have biased the current conclusions, implying that more studies are needed to confirm this outcome.

Methionine in the body provides methyl groups through a transmethyl action, simultaneously producing SAH, which is further transformed into Hcy. Hcy can accept the methyl group supplied by N-CH3-FH4 and regenerate Met, forming a cyclic process known as the Met cycle. AD acts as a chronic neurodegenerative disease, and it has been shown that its pathogenesis is correlated with changes in DNA or RNA methylation (Wong et al., 2013; Blanch et al., 2016; Stoccoro et al., 2017, 2018; Xu et al., 2019; Han et al., 2020). Met and folate cycles of single-carbon metabolism provide carbon units for SAM required for DNA methylation, which is an intracellular universal methylator (Coppedè, 2015). Met synthase reductase (MTRR) forms a complex with MTR to maintain its activity in an active state (Coppedè, 2015). Once generated, Met is converted to SAM, which is then imported into the mitochondria *via* a mitochondrial vector and used by DNA methylation reactions (DNMTs) for mtDNA methylation reactions (Kishita et al., 2015). Thus, the Met cycle, an important component of DNA/RNA methylation, influences the occurrence of AD.

Homocysteine is an amino acid containing a sulfhydryl group, and its levels are influenced by the B vitamins cobalamin, vitamin B_6_, and folic acid, which act as cofactors for enzymes involved in the Met metabolism (Stabler et al., 1988). The results of this review reveal that Hcy levels in the blood of patients with AD are higher than those in cognitively normal groups. Previous studies are in line with our findings, which show that elevated blood Hcy levels are an independent risk factor for the development of AD. Hcy levels were significantly higher in patients with MCI than in normal controls, and this level was negatively correlated with cognitive function in patients with MCI (Riggs et al., 1996; Ravaglia et al., 2000b; Budge et al., 2002; Duthie et al., 2002; Prins et al., 2002; Clarke et al., 2003). Lauriola et al. (2021) revealed that blood Hcy levels are significantly increased in patients with AD and MCI. In the Oxford Project to Investigate Memory and Ageing (OPIMA) population, higher serum Hcy levels were observed in patients with dementia of Alzheimer’s type and patients with histologically confirmed AD than in controls (Selhub et al., 2010). Such higher Hcy levels are also accompanied by low serum folate and vitamin B_12_ levels (Clarke et al., 1998). This may be related to Hcy being an intermediate product of the one-carbon metabolism and affecting cognitive function by interfering with the DNA/RNA methylation process, resulting in neurodegenerative disease. In general, Hcy seems to be a risk factor that affects AD *via* the DNA/RNA methylation pathway, through a series of changes in AD-related substances (Pi et al., 2020).

Vitamin B_12_ is an essential water-soluble micronutrient that must be taken up in sufficient quantities from one’s diet (Sahu et al., 2022). Although our results of the plasma subgroup showed no statistically significant differences in vitamin B12 levels, they are crucial for maintaining neuronal health (Green et al., 2017), and deficiencies are characterized by cognitive and psychiatric disturbances (Morris et al., 2006). Current interest in vitamin B_12_ as a risk factor for AD is based on its relationship as a cofactor in the Hcy metabolism (Morris et al., 2006). A cross-sectional study of 206 patients with AD recruited from six hospitals in China (Qian et al., 2022) and Jakubowski et al.’s (2021) research both indicated a possible pathogenic role of vitamin B_12_ in MCI. Dos Santos and Pardi (2020) also indicated in a case–control study that there is a decrease in the levels of vitamin B_12_ in patients with AD compared to healthy groups with biochemical evaluations. Perhaps due to the anti-oxidant property of vitamin B_12_ (McCaddon, 2013). An important anti-oxidant mode of action of vitamin B_12_ is closely linked to AD: a reduction in Hcy-induced oxidative stress (Sahu et al., 2022). Vitamin B_12_ is an important cofactor of MTR, which converts Hcy into Met. Vitamin B_12_ deficiency reduces the conversion of Hcy to Met, leading to elevated intracellular Hcy levels (Green et al., 2017). Thus, it is involved in the one-carbon metabolism, reducing the generation of carbon units necessary for DNA/RNA methylation and accelerating the occurrence and development of AD. Our meta-analysis do not show a clear association between AD/MCI and vitamin B_12_ levels, possibly due to large heterogeneities and publication biases, or to the large gap in publication times, but they provide a new entry point for studying the relationship between the class of B-vitamins and AD/MCI in future.

*S-*adenosylmethionine is synthesized from dietary *L*-methionine by the enzyme Met adenosyltransferase, often considered an intermediary metabolite in the folate and B_12_-dependent Met cycle, and plays pivotal roles in cellular biochemistry (Shea and Chan, 2008). Through a qualitative analysis, we found that high SAM levels might be risk factors for the occurrence of AD. A study on the relationship between SAM and the polyunsaturated fatty acid metabolism indicated a significant increase in the plasma concentrations of Hcy, SAM, and SAH among patients with AD compared to that among control subjects, and the SAM/SAH ratio was lower in Alzheimer’s group than in the control group (Selley, 2007). Furthermore, Shea and Chan (2008) research indicated that SAM supplementation alleviates a wide range of factors that contribute to the manifestation of neurodegeneration in AD, ranging from antecedent/early events such as increased oxidative stress and genetic predispositions to later manifestations of neurodegeneration such as loss of cognitive performance and increased aggression.

Methionine is an essential amino acid obtained from one’s diet. In addition to being required for protein biosynthesis, Met is metabolized to generate metabolites that play key roles in a number of cellular functions (Dash et al., 2016). Although few studies have focused on blood Met levels in humans, some associations have been shown between rat models of cognitive impairment and Met levels (Alzoubi et al., 2022; Khodir et al., 2022). Met is the sole substrate of Met adenosine transferase (MAT) for SAM synthesis, whereas SAM is a universal methyl donor for various methyl transfer reactions, including the methylation of DNA, RNA, and neurotransmitters in all organisms (Selhub et al., 2010). One study concluded that the Hcy-Met cycle is a metabolic sensor system that mediates receptor-independent metabolism-associated pathological signal identification and the regulation of methylation (Shen et al., 2020). Meanwhile, it has been shown that the onset of AD is associated with methylation, whereas, Balmik and Chinnathambi (2021) suggested that methylation was a key regulator of Tau aggregation and neuronal health in AD. This systematic review and meta-analysis focused on the components of the Met cycle that are involved in AD/MCI. However, further research is needed to determine the changes in blood levels of methylation components in patients with AD/MCI, such as SAM, SAH, or the ratio of SAM/SAH. Perhaps for some reasons, there is less research in this field, and randomized controlled experiments of humans are also rare, which will be an exploration aspect for future research.

## Clinical implications

As a chronic degenerative neuropathy disease, AD has a slow and insidious onset. With the continuous increase in the degree of aging, the incidence of AD is also increasing, placing a huge burden on families and the society. This meta-analysis explored the potential relationship between AD/MCI and components of the Met cycle. This provides a new approach for diagnosis and intervention before the onset of AD.

## Advantages and limitations

Our study has some potential limitations. First, all included studies were observational, and the level of evidence was somewhat inadequate; therefore, causal relationship or association could not be established. Second, there is a significant association of AD/MCI with the Hcy or vitamin B12 level; however, due to the very low GRADE level of evidence, caution is required when interpreting our results. Meanwhile, the number of cases of blood vitamin B_12_ and Hcy level evaluations in patients with MCI was small, and the results may be controversial. Third, SAM and SAH were not discussed further because of the small number of studies on them, which has implications for this study. Moreover, heterogeneity was found in some of these comparisons, and it is difficult to derive the underlying cause of concordance because of the presence of confounding variables. Finally, the study populations we included comprise different AD types, and we did not perform subgroup analyses due to inclusion literature restrictions but directly combined the data; such subgroup analyses could be a possible direction for future research.

Despite these limitations, this study has several strengths. First, we searched four major databases, namely, PubMed, Embase, Cochrane Library, and Web of Science, and manually retrieved the reference lists of possible articles, ensuring that the included literature was comprehensive and accurate. Second, the quality of the included studies was checked to ensure that only extensive, detailed, and high-quality literature was included. Third, this review synthesizes qualitative and quantitative data on the blood levels of circulating Met components in patients with AD/MCI, which makes our results even more convincing. Finally, we explored potential sources of heterogeneity through a sensitivity analysis and excluded articles individually, which ensures that our study results are reliable and robust.

## Conclusion

This systematic review and meta-analysis confirms that circulating Met components affect patients with AD compared to cognitively normal individuals, with patients exhibiting higher blood Hcy levels. The results of a qualitative analysis also indicate that high Met and SAM levels are risk factors for AD, which supports the association between Met cycle components and AD/MCI. Further investigations of these indices as potential biomarkers for early diagnosis and interventions for AD/MCI are warranted.

## Data availability statement

The original contributions presented in this study are included in the article/Supplementary Material, further inquiries can be directed to the corresponding authors.

## Author contributions

YZ and HC contributed to choose the topic and study design. XD, SM, BC, and YZ searched the literature. XG, JZ, and HW contributed to the data collection. YZZ, XD, and BC performed statistical analyses and interpretation of results. YZ, XD, and FG drafted the manuscript and edited the language. SL and HC contributed to the critical revision of the manuscript and had primary responsibility for the final content. All authors participated in the critical revisions and approved the final version of the manuscript.

## Conflict of interest

The authors declare that the research was conducted in the absence of any commercial or financial relationships that could be construed as a potential conflict of interest.

## Publisher’s note

All claims expressed in this article are solely those of the authors and do not necessarily represent those of their affiliated organizations, or those of the publisher, the editors and the reviewers. Any product that may be evaluated in this article, or claim that may be made by its manufacturer, is not guaranteed or endorsed by the publisher.

## Footnotes

1
https://jbi.global/critical-appraisal-tools

2
https://gdt.gradepro.org/app/#projects

## Supplementary material

Supplementary Figure 1 Funnel plot with pseudo 95% confidence limits of 15 studies comparing blood Hcy levels between AD vs. Control.

Supplementary Figure 2 Funnel plot with pseudo 95% confidence limits of 11 studies comparing blood vitamin B_12_ levels between AD vs. Control.

## Abbreviations

AD: Alzheimer’s disease
CAMCOG: Cambridge cognition examination
CSF: cerebrospinal fluid
CI: confidence interval
Cys: cysteine
DNMTs: DNA methylation reactions
Hcy: homocysteine
IQRs: interquartile ranges
JBI: Joanna Briggs Institute
MAT: Met adenosine transferase
Met: methionine
MTR: methionine synthase
MTRR: methionine synthase reductase
MTHFR: methylenetetrahydrofolate reductase
MCI: mild cognitive impairment
MMSE: mini-mental state examination
OPIMA: Oxford project to investigate memory and aging
RFC-1: reduced folate carrier
SAH: *S*-adenosylhomocysteine
SAM: *S*-adenosylmethionine
SD: standard deviation
SE: standard error
SMDs: standardized mean differences.

## References

[B1] Altuna-Azkargorta M. Mendioroz-Iriarte M. (2018). Blood biomarkers in Alzheimer’s disease. *Neurologia* S0213-4853(18)30091-4. 10.1016/j.nrl.2018.03.006 [Epub ahead of print]. 29752036

[B2] Alzoubi K. H. Khabour O. F. Alfaqih M. Tashtoush M. Al-Azzam S. I. Mhaidat N. M. (2022). The protective effects of pioglitazone against cognitive impairment caused by L-methionine administration in a rat model. *CNS Neurol. Disord. Drug Targets* 21 77–84. 10.2174/1871527320666210809122523 34370649

[B3] Annerbo S. Kivipelto M. Lokk J. (2009). A prospective study on the development of Alzheimer’s disease with regard to thyroid-stimulating hormone and homocysteine. *Dement. Geriatr. Cogn. Disord.* 28 275–280. 10.1159/000242439 19797897

[B4] Annerbo S. Wahlund L. O. Lökk J. (2005). The relation between homocysteine levels and development of Alzheimer’s disease in mild cognitive impairment patients. *Dement. Geriatr. Cogn. Disord.* 20 209–214. 10.1159/000087297 16088136

[B5] Annerbo S. Wahlund L. O. Lökk J. (2006). The significance of thyroid-stimulating hormone and homocysteine in the development of Alzheimer’s disease in mild cognitive impairment: a 6-year follow-up study. *Am. J. Alzheimers Dis. Other Demen.* 21 182–188. 10.1177/1533317506289282 16869339 PMC10833276

[B6] Atkins D. Best D. Briss P. A. Eccles M. Falck-Ytter Y. Flottorp S. (2004). Grading quality of evidence and strength of recommendations. *BMJ* 328:1490. 10.1136/bmj.328.7454.1490 15205295 PMC428525

[B7] Balmik A. A. Chinnathambi S. (2021). Methylation as a key regulator of tau aggregation and neuronal health in Alzheimer’s disease. *Cell Commun. Signal.* 19:51. 10.1186/s12964-021-00732-z 33962636 PMC8103764

[B8] Bednarska-Makaruk M. Graban A. Sobczyńska-Malefora A. Harrington D. J. Mitchell M. Voong K. (2016). Homocysteine metabolism and the associations of global DNA methylation with selected gene polymorphisms and nutritional factors in patients with dementia. *Exp. Gerontol.* 81 83–91. 10.1016/j.exger.2016.05.002 27167582

[B9] Blanch M. Mosquera J. L. Ansoleaga B. Ferrer I. Barrachina M. (2016). Altered mitochondrial DNA methylation pattern in Alzheimer disease-related pathology and in Parkinson disease. *Am. J. Pathol.* 186 385–397. 10.1016/j.ajpath.2015.10.004 26776077

[B10] Blasko I. Jellinger K. Kemmler G. Krampla W. Jungwirth S. Wichart I. (2008). Conversion from cognitive health to mild cognitive impairment and Alzheimer’s disease: prediction by plasma amyloid beta 42, medial temporal lobe atrophy and homocysteine. *Neurobiol. Aging* 29 1–11. 10.1016/j.neurobiolaging.2006.09.002 17055615

[B11] Budge M. M. de Jager C. Hogervorst E. Smith A. D. (2002). Total plasma homocysteine, age, systolic blood pressure, and cognitive performance in older people. *J. Am. Geriatr. Soc.* 50 2014–2018. 10.1046/j.1532-5415.2002.50614.x 12473014

[B12] Budge M. Johnston C. Hogervorst E. de Jager C. Milwain E. Iversen S. D. (2000). Plasma total homocysteine and cognitive performance in a volunteer elderly population. *Ann. N. Y. Acad. Sci.* 903 407–410. 10.1111/j.1749-6632.2000.tb06392.x 10818531

[B13] Cankurtaran M. Yesil Y. Kuyumcu M. E. Oztürk Z. A. Yavuz B. B. Halil M. (2013). Altered levels of homocysteine and serum natural antioxidants links oxidative damage to Alzheimer’s disease. *J. Alzheimers Dis.* 33 1051–1058. 10.3233/JAD-2012-121630 23109556

[B14] Cervellati C. Romani A. Seripa D. Cremonini E. Bosi C. Magon S. (2014). Oxidative balance, homocysteine, and uric acid levels in older patients with late onset Alzheimer’s disease or vascular dementia. *J. Neurol. Sci.* 337 156–161. 10.1016/j.jns.2013.11.041 24321755

[B15] Chandra A. Dervenoulas G. Politis M. (2019). Magnetic resonance imaging in Alzheimer’s disease and mild cognitive impairment. *J. Neurol.* 266 1293–1302. 10.1007/s00415-018-9016-3 30120563 PMC6517561

[B16] Chiang P. K. Gordon R. K. Tal J. Zeng G. C. Doctor B. P. Pardhasaradhi K. (1996). S-Adenosylmethionine and methylation. *Faseb J.* 10 471–480. 10.1096/fasebj.10.4.8647346 8647346

[B17] Clarke R. Harrison G. Richards S. (2003). Effect of vitamins and aspirin on markers of platelet activation, oxidative stress and homocysteine in people at high risk of dementia. *J. Intern. Med.* 254 67–75. 10.1046/j.1365-2796.2003.01154.x 12823643

[B18] Clarke R. Smith A. D. Jobst K. A. Refsum H. Sutton L. Ueland P. M. (1998). Folate, vitamin B12, and serum total homocysteine levels in confirmed Alzheimer disease. *Arch. Neurol.* 55 1449–1455. 10.1001/archneur.55.11.1449 9823829

[B19] Coppedè F. (2015). The genetics of folate metabolism and maternal risk of birth of a child with down syndrome and associated congenital heart defects. *Front. Genet.* 6:223. 10.3389/fgene.2015.00223 26161087 PMC4479818

[B20] Corso G. Cristofano A. Sapere N. la Marca G. Angiolillo A. Vitale M. (2017). Serum amino acid profiles in normal subjects and in patients with or at risk of Alzheimer dementia. *Dement. Geriatr. Cogn. Dis. Extra* 7 143–159. 10.1159/000466688 28626469 PMC5471778

[B21] Dash P. K. Hergenroeder G. W. Jeter C. B. Choi H. A. Kobori N. Moore A. N. (2016). Traumatic brain injury alters methionine metabolism: implications for pathophysiology. *Front. Syst. Neurosci.* 10:36. 10.3389/fnsys.2016.00036 27199685 PMC4850826

[B22] Dayon L. Guiraud S. P. Corthésy J. Da Silva L. Migliavacca E. Tautvydaitë D. (2017). One-carbon metabolism, cognitive impairment and CSF measures of Alzheimer pathology: homocysteine and beyond. *Alzheimers Res. Ther.* 9:43. 10.1186/s13195-017-0270-x 28623948 PMC5473969

[B23] de Leeuw F. A. Tijms B. M. Doorduijn A. S. Hendriksen H. M. A. van de Rest O. de van der Schueren M. A. E. (2020a). LDL cholesterol and uridine levels in blood are potential nutritional biomarkers for clinical progression in Alzheimer’s disease: the NUDAD project. *Alzheimers Dement.* 12:e12120. 10.1002/dad2.12120 PMC7772937 33392381

[B24] de Leeuw F. A. van der Flier W. M. Tijms B. M. Scheltens P. Mendes V. M. Manadas B. (2020b). Specific nutritional biomarker profiles in mild cognitive impairment and subjective cognitive decline are associated with clinical progression: the NUDAD project. *J. Am. Med. Dir. Assoc.* 21 1513.e1–1513.e17. 10.1016/j.jamda.2019.12.009 32001171

[B25] DeMarshall C. A. Nagele E. P. Sarkar A. Acharya N. K. Godsey G. Goldwaser E. L. (2016). Detection of Alzheimer’s disease at mild cognitive impairment and disease progression using autoantibodies as blood-based biomarkers. *Alzheimers Dement.* 3 51–62. 10.1016/j.dadm.2016.03.002 27239548 PMC4879649

[B26] Devčić S. Glamuzina L. Ruljancic N. Mihanovic M. (2014). There are no differences in IL-6, CRP and homocystein concentrations between women whose mothers had AD and women whose mothers did not have AD. *Psychiatry Res.* 220 970–974. 10.1016/j.psychres.2014.08.059 25240941

[B27] Dos Santos G. A. A. Pardi P. C. (2020). Biomarkers in Alzheimer’s disease: evaluation of platelets, hemoglobin and vitamin B12. *Dement. Neuropsychol.* 14 35–40. 10.1590/1980-57642020dn14-010006 32206196 PMC7077854

[B28] Dubois B. Feldman H. H. Jacova C. Hampel H. Molinuevo J. L. Blennow K. (2014). Advancing research diagnostic criteria for Alzheimer’s disease: the IWG-2 criteria. *Lancet Neurol.* 13 614–629. 10.1016/S1474-4422(14)70090-0 24849862

[B29] Duthie S. J. Whalley L. J. Collins A. R. Leaper S. Berger K. Deary I. J. (2002). Homocysteine, B vitamin status, and cognitive function in the elderly. *Am. J. Clin. Nutr.* 75 908–913. 10.1093/ajcn/75.5.908 11976166

[B30] Faux N. G. Ellis K. A. Porter L. Fowler C. J. Laws S. M. Martins R. N. (2011). Homocysteine, vitamin B12, and folic acid levels in Alzheimer’s disease, mild cognitive impairment, and healthy elderly: baseline characteristics in subjects of the Australian imaging biomarker lifestyle STUDY. *J. Alzheimers Dis.* 27 909–922. 10.3233/JAD-2011-110752 21891867

[B31] Frick B. Gruber B. Schroecksnadel K. Leblhuber F. Fuchs D. (2006). Homocysteine but not neopterin declines in demented patients on B vitamins. *J. Neural Transm. (Vienna)* 113 1815–1819. 10.1007/s00702-006-0539-x 16988797

[B32] Green R. Allen L. H. Bjørke-Monsen A. L. Brito A. Guéant J. L. Miller J. W. (2017). Vitamin B_(12)_ deficiency. *Nat. Rev. Dis. Primers* 3:17040. 10.1038/nrdp.2017.40 28660890

[B33] Grossi E. Stoccoro A. Tannorella P. Migliore L. Coppedè F. (2016). Artificial neural networks link one-carbon metabolism to gene-promoter methylation in Alzheimer’s disease. *J. Alzheimers Dis.* 53 1517–1522. 10.3233/JAD-160210 27392858

[B34] Han M. Liu Z. Xu Y. Liu X. Wang D. Li F. (2020). Abnormality of m6A mRNA methylation is involved in Alzheimer’s disease. *Front. Neurosci.* 14:98. 10.3389/fnins.2020.00098 32184705 PMC7058666

[B35] Hooshmand B. Refsum H. Smith A. D. Kalpouzos G. Mangialasche F. von Arnim C. A. F. (2019). Association of methionine to homocysteine status with brain magnetic resonance imaging measures and risk of dementia. *JAMA Psychiatry* 76 1198–1205. 10.1001/jamapsychiatry.2019.1694 31339527 PMC6659152

[B36] Jakubowski H. Zioła-Frankowska A. Frankowski M. Perła-Kaján J. Refsum H. de Jager C. A. (2021). B vitamins prevent iron-associated brain atrophy and domain-specific effects of iron, copper, aluminum, and silicon on cognition in mild cognitive impairment. *J. Alzheimers Dis.* 84 1039–1055. 10.3233/JAD-215085 34602484 PMC8673493

[B37] James B. D. Wilson R. S. Boyle P. A. Trojanowski J. Q. Bennett D. A. Schneider J. A. (2016). TDP-43 stage, mixed pathologies, and clinical Alzheimer’s-type dementia. *Brain* 139 2983–2993. 10.1093/brain/aww224 27694152 PMC5091047

[B38] Kennedy B. P. Bottiglieri T. Arning E. Ziegler M. G. Hansen L. A. Masliah E. (2004). Elevated S-adenosylhomocysteine in Alzheimer brain: influence on methyltransferases and cognitive function. *J. Neural Transm. (Vienna)* 111 547–567. 10.1007/s00702-003-0096-5 15057524

[B39] Khodir S. A. Faried M. A. Abd-Elhafiz H. I. Sweed E. M. (2022). Sitagliptin attenuates the cognitive deficits in L-methionine-induced vascular dementia in rats. *Biomed Res. Int.* 2022:7222590. 10.1155/2022/7222590 35265716 PMC8898801

[B40] Kishita Y. Pajak A. Bolar N. A. Marobbio C. M. Maffezzini C. Miniero D. V. (2015). Intra-mitochondrial methylation deficiency due to mutations in SLC25A26. *Am. J. Hum. Genet.* 97 761–768. 10.1016/j.ajhg.2015.09.013 26522469 PMC4667130

[B41] Lam A. B. Kervin K. Tanis J. E. (2021). Vitamin B_(12)_ impacts amyloid beta-induced proteotoxicity by regulating the methionine/S-adenosylmethionine cycle. *Cell Rep.* 36:109753. 10.1016/j.celrep.2021.109753 34592146 PMC8522492

[B42] Lauriola M. D’Onofrio G. Ciccone F. Germano C. Cascavilla L. Paris F. (2021). Relationship of homocysteine plasma levels with mild cognitive impairment, Alzheimer’s disease, vascular dementia, psychobehavioral, and functional complications. *J. Alzheimers Dis.* 82 235–248. 10.3233/JAD-210166 34057086 PMC8293649

[B43] Lehmann M. Gottfries C. G. Regland B. (1999). Identification of cognitive impairment in the elderly: homocysteine is an early marker. *Dement. Geriatr. Cogn. Disord.* 10 12–20. 10.1159/000017092 9844033

[B44] Li Q. Cui J. Fang C. Liu M. Min G. Li L. (2017). S-Adenosylmethionine attenuates oxidative stress and neuroinflammation induced by amyloid-β through modulation of glutathione metabolism. *J. Alzheimers Dis.* 58 549–558. 10.3233/JAD-170177 28453493

[B45] Linnebank M. Popp J. Smulders Y. Smith D. Semmler A. Farkas M. (2010). S-adenosylmethionine is decreased in the cerebrospinal fluid of patients with Alzheimer’s disease. *Neurodegener. Dis.* 7 373–378. 10.1159/000309657 20523031

[B46] McCaddon A. (2013). Vitamin B12 in neurology and ageing; clinical and genetic aspects. *Biochimie* 95 1066–1076. 10.1016/j.biochi.2012.11.017 23228515

[B47] McCaddon A. Davies G. Hudson P. Tandy S. Cattell H. (1998). Total serum homocysteine in senile dementia of Alzheimer type. *Int. J. Geriatr. Psychiatry* 13 235–239. 10.1002/(SICI)1099-1166(199804)13:4<235::AID-GPS761>3.0.CO;2-8 9646150

[B48] McCaddon A. Hudson P. Davies G. Hughes A. Williams J. H. Wilkinson C. (2001). Homocysteine and cognitive decline in healthy elderly. *Dement. Geriatr. Cogn. Disord.* 12 309–313. 10.1159/000051275 11455131

[B49] Mecca A. P. van Dyck C. H. (2021). Alzheimer’s & dementia: the journal of the Alzheimer’s association. *Alzheimers Dement.* 17 316–317. 10.1002/alz.12190 33047474 PMC7962853

[B50] Miller J. W. Green R. Mungas D. M. Reed B. R. Jagust W. J. (2002). Homocysteine, vitamin B6, and vascular disease in AD patients. *Neurology* 58 1471–1475. 10.1212/WNL.58.10.1471 12034781

[B51] Moola S. Munn Z. Tufanaru C. Aromataris E. Sears K. Sfetic R. (2019). “Chapter 7: systematic reviews of etiology and risk,” in *JBI Manual for Evidence Synthesis*, eds Aromataris E. Munn Z. (Adelaide, SA: JBI). 10.46658/JBIRM-17-06

[B52] Morris M. C. Schneider J. A. Tangney C. C. (2006). Thoughts on B-vitamins and dementia. *J. Alzheimers Dis.* 9 429–433. 10.3233/JAD-2006-9409 16917152 PMC3428233

[B53] Munn Z. Barker T. H. Moola S. Tufanaru C. Stern C. McArthur A. (2020). Methodological quality of case series studies: an introduction to the JBI critical appraisal tool. *JBI Evid. Synth.* 18 2127–2133. 10.11124/JBISRIR-D-19-00099 33038125

[B54] O’Leary F. Allman-Farinelli M. Samman S. (2012). Vitamin B12 status, cognitive decline and dementia: a systematic review of prospective cohort studies. *Br. J. Nutr.* 108 1948–1961. 10.1017/S0007114512004175 23084026

[B55] Page M. J. McKenzie J. E. Bossuyt P. M. Boutron I. Hoffmann T. C. Mulrow C. D. (2021). The PRISMA 2020 statement: an updated guideline for reporting systematic reviews. *BMJ* 372:n71. 10.1136/bmj.n71 33782057 PMC8005924

[B56] Panza F. Frisardi V. Capurso C. D’Introno A. Colacicco A. M. Di Palo A. (2009). Polyunsaturated fatty acid and S-adenosylmethionine supplementation in predementia syndromes and Alzheimer’s disease: a review. *ScientificWorldJournal* 9 373–389. 10.1100/tsw.2009.48 19468660 PMC5823128

[B57] Petersen R. C. (2009). Early diagnosis of Alzheimer’s disease: is MCI too late? *Curr. Alzheimer Res.* 6 324–330. 10.2174/156720509788929237 19689230 PMC3098139

[B58] Pi T. Liu B. Shi J. (2020). Abnormal homocysteine metabolism: an insight of Alzheimer’s disease from DNA methylation. *Behav. Neurol.* 2020:8438602. 10.1155/2020/8438602 32963633 PMC7495165

[B59] Pi T. Wei S. Jiang Y. Shi J. S. (2021). High methionine diet-induced Alzheimer’s disease like symptoms are accompanied by 5-methylcytosine elevated levels in the brain. *Behav. Neurol.* 2021:6683318. 10.1155/2021/6683318 33880134 PMC8046555

[B60] Piazza F. Galimberti G. Conti E. Isella V. Perlangeli M. V. Speranza T. (2012). Increased tissue factor pathway inhibitor and homocysteine in Alzheimer’s disease. *Neurobiol. Aging* 33 226–233. 10.1016/j.neurobiolaging.2010.02.016 20359777

[B61] Prins N. D. Den Heijer T. Hofman A. Koudstaal P. J. Jolles J. Clarke R. (2002). Homocysteine and cognitive function in the elderly: the rotterdam scan study. *Neurology* 59 1375–1380. 10.1212/01.WNL.0000032494.05619.93 12427887

[B62] Qian T. Zhao L. Pan X. Sang S. Xu Y. Wang C. (2022). Association between blood biochemical factors contributing to cognitive decline and B vitamins in patients with Alzheimer’s disease. *Front. Nutr.* 9:823573. 10.3389/fnut.2022.823573 35265656 PMC8898888

[B63] Raszewski G. Chwedorowicz R. Chwedorowicz A. Gustaw Rothenberg K. (2016). Homocysteine, antioxidant vitamins and lipids as biomarkers of neurodegeneration in Alzheimer’s disease versus non-Alzheimer’s dementia. *Ann. Agric. Environ. Med.* 23 193–196. 10.5604/12321966.1196878 27007541

[B64] Ravaglia G. Forti P. Maioli F. Vettori C. Grossi G. Bargossi A. M. (2000a). Elevated plasma homocysteine levels in centenarians are not associated with cognitive impairment. *Mech. Ageing Dev.* 121 251–261. 10.1016/S0047-6374(00)00221-9 11164478

[B65] Ravaglia G. Forti P. Maioli F. Zanardi V. Dalmonte E. Grossi G. (2000b). Blood homocysteine and vitamin B levels are not associated with cognitive skills in healthy normally ageing subjects. *J. Nutr. Health Aging* 4 218–222. 11115804

[B66] Riggs K. M. Spiro A. III Tucker K. Rush D. (1996). Relations of vitamin B-12, vitamin B-6, folate, and homocysteine to cognitive performance in the normative aging study. *Am. J. Clin. Nutr.* 63 306–314. 10.1093/ajcn/63.3.306 8602585

[B67] Ritchie C. Smailagic N. Noel-Storr A. H. Ukoumunne O. Ladds E. C. Martin S. (2017). CSF tau and the CSF tau/ABeta ratio for the diagnosis of Alzheimer’s disease dementia and other dementias in people with mild cognitive impairment (MCI). *Cochrane Database Syst. Rev.* 3:Cd010803. 10.1002/14651858.CD010803.pub2 28328043 PMC6464349

[B68] Román G. C. (2015). MTHFR gene mutations: a potential marker of late-onset Alzheimer’s disease? *J. Alzheimers Dis.* 47 323–327. 10.3233/JAD-150304 26401555

[B69] Román G. C. Mancera-Páez O. Bernal C. (2019). Epigenetic factors in late-onset Alzheimer’s disease: MTHFR and CTH Gene polymorphisms, metabolic transsulfuration and methylation pathways, and B vitamins. *Int. J. Mol. Sci.* 20:319. 10.3390/ijms20020319 PMC6359124 30646578

[B70] Sahu P. Thippeswamy H. Chaturvedi S. K. (2022). Neuropsychiatric manifestations in vitamin B12 deficiency. *Vitam. Horm.* 119 457–470. 10.1016/bs.vh.2022.01.001 35337631

[B71] Selhub J. Troen A. Rosenberg I. H. (2010). B vitamins and the aging brain. *Nutr. Rev.* 68 Suppl 2 S112–S118. 10.1111/j.1753-4887.2010.00346.x 21091944

[B72] Selley M. L. (2004). Increased homocysteine and decreased adenosine formation in Alzheimer’s disease. *Neurol. Res.* 26 554–557. 10.1179/016164104225016182 15265273

[B73] Selley M. L. (2007). A metabolic link between S-adenosylhomocysteine and polyunsaturated fatty acid metabolism in Alzheimer’s disease. *Neurobiol. Aging* 28 1834–1839. 10.1016/j.neurobiolaging.2006.08.003 16996649

[B74] Seshadri S. Beiser A. Selhub J. Jacques P. F. Rosenberg I. H. D’Agostino R. B. (2002). Plasma homocysteine as a risk factor for dementia and Alzheimer’s disease. *N. Engl. J. Med.* 346 476–483. 10.1056/NEJMoa011613 11844848

[B75] Sharma A. Gerbarg P. Bottiglieri T. Massoumi L. Carpenter L. L. Lavretsky H. (2017). S-Adenosylmethionine (SAMe) for neuropsychiatric disorders: a clinician-oriented review of research. *J. Clin. Psychiatry* 78 e656–e667. 10.4088/JCP.16r11113 28682528 PMC5501081

[B76] Shea T. B. Chan A. (2008). S-adenosyl methionine: a natural therapeutic agent effective against multiple hallmarks and risk factors associated with Alzheimer’s disease. *J. Alzheimers Dis.* 13 67–70. 10.3233/JAD-2008-13107 18334758

[B77] Shen W. Gao C. Cueto R. Liu L. Fu H. Shao Y. (2020). Homocysteine-methionine cycle is a metabolic sensor system controlling methylation-regulated pathological signaling. *Redox Biol.* 28:101322. 10.1016/j.redox.2019.101322 31605963 PMC6812029

[B78] Shrubsole M. J. Wagner C. Zhu X. Hou L. Loukachevitch L. V. Ness R. M. (2015). Associations between S-adenosylmethionine, S-adenosylhomocysteine, and colorectal adenoma risk are modified by sex. *Am. J. Cancer Res.* 5 458–465. 25628954 PMC4300688

[B79] Singh M. Prakash A. (2017). Possible role of endothelin receptor against hyperhomocysteinemia and β-amyloid induced AD type of vascular dementia in rats. *Brain Res. Bull.* 133 31–41. 10.1016/j.brainresbull.2017.02.012 28274813

[B80] Sklirou E. Lichter-Konecki U. (2018). Inborn errors of metabolism with cognitive impairment: metabolism defects of phenylalanine, homocysteine and methionine, purine and pyrimidine, and creatine. *Pediatr. Clin. North Am.* 65 267–277. 10.1016/j.pcl.2017.11.009 29502913

[B81] Smith A. D. Refsum H. Bottiglieri T. Fenech M. Hooshmand B. McCaddon A. (2018). Homocysteine and dementia: an international consensus statement. *J. Alzheimers Dis.* 62 561–570. 10.3233/JAD-171042 29480200 PMC5836397

[B82] Stabler S. P. Marcell P. D. Podell E. R. Allen R. H. Savage D. G. Lindenbaum J. (1988). Elevation of total homocysteine in the serum of patients with cobalamin or folate deficiency detected by capillary gas chromatography-mass spectrometry. *J. Clin. Invest.* 81 466–474. 10.1172/JCI113343 3339129 PMC329593

[B83] Stewart R. Asonganyi B. Sherwood R. (2002). Plasma homocysteine and cognitive impairment in an older British African-caribbean population. *J. Am. Geriatr. Soc.* 50 1227–1232. 10.1046/j.1532-5415.2002.50309.x 12133017

[B84] Stoccoro A. Mosca L. Carnicelli V. Cavallari U. Lunetta C. Marocchi A. (2018). Mitochondrial DNA copy number and D-loop region methylation in carriers of amyotrophic lateral sclerosis gene mutations. *Epigenomics* 10 1431–1443. 10.2217/epi-2018-0072 30088417

[B85] Stoccoro A. Siciliano G. Migliore L. Coppedè F. (2017). Decreased methylation of the mitochondrial D-loop region in late-onset Alzheimer’s disease. *J. Alzheimers Dis.* 59 559–564. 10.3233/JAD-170139 28655136

[B86] Tangney C. C. Tang Y. Evans D. A. Morris M. C. (2009). Biochemical indicators of vitamin B12 and folate insufficiency and cognitive decline. *Neurology* 72 361–367. 10.1212/01.wnl.0000341272.48617.b0 19171834 PMC2677500

[B87] Tannorella P. Stoccoro A. Tognoni G. Petrozzi L. Salluzzo M. G. Ragalmuto A. (2015). Methylation analysis of multiple genes in blood DNA of Alzheimer’s disease and healthy individuals. *Neurosci. Lett.* 600 143–147. 10.1016/j.neulet.2015.06.009 26079324

[B88] Troesch B. Weber P. Mohajeri M. H. (2016). Potential links between impaired one-carbon metabolism due to polymorphisms, inadequate B-vitamin status, and the development of Alzheimer’s disease. *Nutrients* 8:803. 10.3390/nu8120803 27973419 PMC5188458

[B89] Veryard L. Jones E. Weaving G. Smith E. Cheek L. Wickramasinghe A. (2013). Pro-inflammatory cytokines IL-1β and TNF-α are not associated with plasma homocysteine concentration in Alzheimer’s disease. *Curr. Alzheimer Res.* 10 174–179. 10.2174/1567205011310020007 23463936

[B90] Wan X. Wang W. Liu J. Tong T. (2014). Estimating the sample mean and standard deviation from the sample size, median, range and/or interquartile range. *BMC Med. Res. Methodol.* 14:135. 10.1186/1471-2288-14-135 25524443 PMC4383202

[B91] Wang Q. Zhao J. Chang H. Liu X. Zhu R. (2021). Homocysteine and folic acid: risk factors for Alzheimer’s disease-an updated meta-analysis. *Front. Aging Neurosci.* 13:665114. 10.3389/fnagi.2021.665114 34122042 PMC8188894

[B92] Wong M. Gertz B. Chestnut B. A. Martin L. J. (2013). Mitochondrial DNMT3A and DNA methylation in skeletal muscle and CNS of transgenic mouse models of ALS. *Front. Cell. Neurosci.* 7:279. 10.3389/fncel.2013.00279 24399935 PMC3872319

[B93] Xu Y. Xu L. Han M. Liu X. Li F. Zhou X. (2019). Altered mitochondrial DNA methylation and mitochondrial DNA copy number in an APP/PS1 transgenic mouse model of Alzheimer disease. *Biochem. Biophys. Res. Commun.* 520 41–46. 10.1016/j.bbrc.2019.09.094 31564416

[B94] Yu L. Chen Y. Wang W. Xiao Z. Hong Y. (2016). Multi-vitamin B supplementation reverses hypoxia-induced tau hyperphosphorylation and improves memory function in adult mice. *J. Alzheimers Dis.* 54 297–306. 10.3233/JAD-160329 27497480

[B95] Zhang D. M. Ye J. X. Mu J. S. Cui X. P. (2017). Efficacy of vitamin B supplementation on cognition in elderly patients with cognitive-related diseases. *J. Geriatr. Psychiatry Neurol.* 30 50–59. 10.1177/0891988716673466 28248558

