# Supplementary material for: Blood levels of circulating methionine components in Alzheimer’s disease and mild cognitive impairment: A systematic review and meta-analysis
Source: Front Aging Neurosci. 2022 Jul 22;14:934070. doi: 10.3389/fnagi.2022.934070 (PMC9354989; doi:10.3389/fnagi.2022.934070)
Supplement: Supplementary file 1 [file Data_Sheet_1.doc]

**Supplementary file 1. Study design-specific critical appraisal tools**

**JBI Case Control Studies Critical Appraisal Tool**

Q1. Were the groups comparable other than presence of disease in cases or absence of disease in controls?

The control group should be representative of the source population that produced the cases. This is usually done by individual matching; wherein controls are selected for each case on the basis of similarity with respect to certain characteristics other than the exposure of interest. Frequency or group matching is an alternative method. Selection bias may result if the groups are not comparable.

Q2. Were cases and controls matched appropriately?

As in item 1, the study should include clear definitions of the source population. Sources from which cases and controls were recruited should be carefully looked at. For example, cancer registries may be used to recruit participants in a study examining risk factors for lung cancer, which typify population-based case control studies. Study participants may be selected from the target population, the source population, or from a pool of eligible participants (such as in hospital-based case control studies).

Q3. Were the same criteria used for identification of cases and controls?

It is useful to determine if patients were included in the study based on either a specified diagnosis or definition. This is more likely to decrease the risk of bias. Characteristics are another useful approach to matching groups, and studies that did not use specified diagnostic methods or definitions should provide evidence on matching by key characteristics. A case should be defined clearly. It is also important that controls must fulfil all the eligibility criteria defined for the cases except for those relating to diagnosis of the disease.

Q4. Was exposure measured in a standard, valid and reliable way?

The study should clearly describe the method of measurement of exposure. Assessing validity requires that a 'gold standard' is available to which the measure can be compared. The validity of exposure measurement usually relates to whether a current measure is appropriate or whether a measure of past exposure is needed.

Case control studies may investigate many different ‘exposures’ that may or may not be associated with the condition. In these cases, reviewers should use the main exposure of interest for their review to answer this question when using this tool at the study level.

Reliability refers to the processes included in an epidemiological study to check repeatability of measurements of the exposures. These usually include intra-observer reliability and inter-observer reliability.

Q5. Was exposure measured in the same way for cases and controls?

As in item 4, the study should clearly describe the method of measurement of exposure. The exposure measures should be clearly defined and described in detail. Assessment of exposure or risk factors should have been carried out according to same procedures or protocols for both cases and controls.

Q6. Were confounding factors identified?

Confounding has occurred where the estimated intervention exposure effect is biased by the presence of some difference between the comparison groups (apart from the exposure investigated/of interest). Typical confounders include baseline characteristics, prognostic factors, or concomitant exposures (e.g. smoking). A confounder is a difference between the comparison groups and it influences the direction of the study results. A high quality study at the level of case control design will identify the potential confounders and measure them (where possible). This is difficult for studies where behavioral, attitudinal or lifestyle factors may impact on the results.

Q7. Were strategies to deal with confounding factors stated?

Strategies to deal with effects of confounding factors may be dealt within the study design or in data analysis. By matching or stratifying sampling of participants, effects of confounding factors can be adjusted for. When dealing with adjustment in data analysis, assess the statistics used in the study. Most will be some form of multivariate regression analysis to account for the confounding factors measured. Look out for a description of statistical methods as regression methods such as logistic regression are usually employed to deal with confounding factors/ variables of interest.

Q8. Were outcomes assessed in a standard, valid and reliable way for cases and controls?

Read the methods section of the paper. If for e.g. lung cancer is assessed based on existing definitions or diagnostic criteria, then the answer to this question is likely to be yes. If lung cancer is assessed using observer reported, or self-reported scales, the risk of over- or under-reporting is increased, and objectivity is compromised. Importantly, determine if the measurement tools used were validated instruments as this has a significant impact on outcome assessment validity.

Having established the objectivity of the outcome measurement (e.g. lung cancer) instrument, it’s important to establish how the measurement was conducted. Were those involved in collecting data trained or educated in the use of the instrument/s? (e.g. radiographers). If there was more than one data collector, were they similar in terms of level of education, clinical or research experience, or level of responsibility in the piece of research being appraised?

Q9. Was the exposure period of interest long enough to be meaningful?

It is particularly important in a case control study that the exposure time was sufficient enough to show an association between the exposure and the outcome. It may be that the exposure period may be too short or too long to influence the outcome.

Q10. Was appropriate statistical analysis used?

As with any consideration of statistical analysis, consideration should be given to whether there was a more appropriate alternate statistical method that could have been used. The methods section should be detailed enough for reviewers to identify which analytical techniques were used (in particular, regression or stratification) and how specific confounders were measured.

For studies utilizing regression analysis, it is useful to identify if the study identified which variables were included and how they related to the outcome. If stratification was the analytical approach used, were the strata of analysis defined by the specified variables? Additionally, it is also important to assess the appropriateness of the analytical strategy in terms of the assumptions associated with the approach as differing methods of analysis are based on differing assumptions about the data and how it will respond.

**JBI Analytical cross-sectional studies Critical Appraisal Tool**

Q1. Were the criteria for inclusion in the sample clearly defined?

The authors should provide clear inclusion and exclusion criteria that they developed prior to recruitment of the study participants. The inclusion/exclusion criteria should be specified (e.g., risk, stage of disease progression) with sufficient detail and all the necessary information critical to the study.

Q2. Were the study subjects and the setting described in detail?

The study sample should be described in sufficient detail so that other researchers can determine if it is comparable to the population of interest to them. The authors should provide a clear description of the population from which the study participants were selected or recruited, including demographics, location, and time period.

Q3. Was the exposure measured in a valid and reliable way?

The study should clearly describe the method of measurement of exposure. Assessing validity requires that a 'gold standard' is available to which the measure can be compared. The validity of exposure measurement usually relates to whether a current measure is appropriate or whether a measure of past exposure is needed.

Reliability refers to the processes included in an epidemiological study to check repeatability of measurements of the exposures. These usually include intra-observer reliability and inter-observer reliability.

Q4. Were objective, standard criteria used for measurement of the condition?

It is useful to determine if patients were included in the study based on either a specified diagnosis or definition. This is more likely to decrease the risk of bias. Characteristics are another useful approach to matching groups, and studies that did not use specified diagnostic methods or definitions should provide evidence on matching by key characteristics

Q5. Were confounding factors identified?

Confounding has occurred where the estimated intervention exposure effect is biased by the presence of some difference between the comparison groups (apart from the exposure investigated/of interest). Typical confounders include baseline characteristics, prognostic factors, or concomitant exposures (e.g. smoking). A confounder is a difference between the comparison groups and it influences the direction of the study results. A high quality study at the level of cohort design will identify the potential confounders and measure them (where possible). This is difficult for studies where behavioral, attitudinal or lifestyle factors may impact on the results.

Q6. Were strategies to deal with confounding factors stated?

Strategies to deal with effects of confounding factors may be dealt within the study design or in data analysis. By matching or stratifying sampling of participants, effects of confounding factors can be adjusted for. When dealing with adjustment in data analysis, assess the statistics used in the study. Most will be some form of multivariate regression analysis to account for the confounding factors measured.

Q7. Were the outcomes measured in a valid and reliable way?

Read the methods section of the paper. If for e.g. lung cancer is assessed based on existing definitions or diagnostic criteria, then the answer to this question is likely to be yes. If lung cancer is assessed using observer reported, or self-reported scales, the risk of over- or under-reporting is increased, and objectivity is compromised. Importantly, determine if the measurement tools used were validated instruments as this has a significant impact on outcome assessment validity.

Having established the objectivity of the outcome measurement (e.g. lung cancer) instrument, it’s important to establish how the measurement was conducted. Were those involved in collecting data trained or educated in the use of the instrument/s? (e.g. radiographers). If there was more than one data collector, were they similar in terms of level of education, clinical or research experience, or level of responsibility in the piece of research being appraised?

Q8. Was appropriate statistical analysis used?

As with any consideration of statistical analysis, consideration should be given to whether there was a more appropriate alternate statistical method that could have been used. The methods section should be detailed enough for reviewers to identify which analytical techniques were used (in particular, regression or stratification) and how specific confounders were measured.

For studies utilizing regression analysis, it is useful to identify if the study identified which variables were included and how they related to the outcome. If stratification was the analytical approach used, were the strata of analysis defined by the specified variables? Additionally, it is also important to assess the appropriateness of the analytical strategy in terms of the assumptions associated with the approach as differing methods of analysis are based on differing assumptions about the data and how it will respond.

**JBI Cohort studies Critical Appraisal Tool**

Q1. Were the two groups similar and recruited from the same population?

Check the paper carefully for descriptions of participants to determine if patients within and across groups have similar characteristics in relation to exposure (e.g. risk factor under investigation). The two groups selected for comparison should be as similar as possible in all characteristics except for their exposure status, relevant to the study in question. The authors should provide clear inclusion and exclusion criteria that they developed prior to recruitment of the study participants.

Q2. Were the exposures measured similarly to assign people to both exposed and unexposed groups?

A high quality study at the level of cohort design should mention or describe how the exposures were measured. The exposure measures should be clearly defined and described in detail. This will enable reviewers to assess whether or not the participants received the exposure of interest.

Q3. Was the exposure measured in a valid and reliable way?

The study should clearly describe the method of measurement of exposure. Assessing validity requires that a 'gold standard' is available to which the measure can be compared. The validity of exposure measurement usually relates to whether a current measure is appropriate or whether a measure of past exposure is needed.

Reliability refers to the processes included in an epidemiological study to check repeatability of measurements of the exposures. These usually include intra-observer reliability and inter-observer reliability.

Q4. Were confounding factors identified?

Confounding has occurred where the estimated intervention exposure effect is biased by the presence of some difference between the comparison groups (apart from the exposure investigated/of interest). Typical confounders include baseline characteristics, prognostic factors, or concomitant exposures (e.g. smoking). A confounder is a difference between the comparison groups and it influences the direction of the study results. A high quality study at the level of cohort design will identify the potential confounders and measure them (where possible). This is difficult for studies where behavioral, attitudinal or lifestyle factors may impact on the results.

Q5. Were strategies to deal with confounding factors stated?

Strategies to deal with effects of confounding factors may be dealt within the study design or in data analysis. By matching or stratifying sampling of participants, effects of confounding factors can be adjusted for. When dealing with adjustment in data analysis, assess the statistics used in the study. Most will be some form of multivariate regression analysis to account for the confounding factors measured. Look out for a description of statistical methods as regression methods such as logistic regression are usually employed to deal with confounding factors/variables of interest.

Q6. Were the groups/participants free of the outcome at the start of the study (or at the moment of exposure)?

The participants should be free of the outcomes of interest at the start of the study. Refer to the ‘methods’ section in the paper for this information, which is usually found in descriptions of participant/sample recruitment, definitions of variables, and/or inclusion/exclusion criteria.

Q7. Were the outcomes measured in a valid and reliable way?

Read the methods section of the paper. If for e.g. lung cancer is assessed based on existing definitions or diagnostic criteria, then the answer to this question is likely to be yes. If lung cancer is assessed using observer reported, or self-reported scales, the risk of over- or under-reporting is increased, and objectivity is compromised. Importantly, determine if the measurement tools used were validated instruments as this has a significant impact on outcome assessment validity.

Having established the objectivity of the outcome measurement (e.g. lung cancer) instrument, it’s important to establish how the measurement was conducted. Were those involved in collecting data trained or educated in the use of the instrument/s? (e.g. radiographers). If there was more than one data collector, were they similar in terms of level of education, clinical or research experience, or level of responsibility in the piece of research being appraised?

Q8. Was the follow up time reported and sufficient to be long enough for outcomes to occur?

The appropriate length of time for follow up will vary with the nature and characteristics of the population of interest and/or the intervention, disease or exposure. To estimate an appropriate duration of follow up, read across multiple papers and take note of the range for duration of follow up. The opinions of experts in clinical practice or clinical research may also assist in determining an appropriate duration of follow up. For example, a longer timeframe may be needed to examine the association between occupational exposure to asbestos and the risk of lung cancer. It is important, particularly in cohort studies that follow up is long enough to enable the outcomes. However, it should be remembered that the research question and outcomes being examined would probably dictate the follow up time.

Q9. Was follow up complete, and if not, were the reasons to loss to follow up described and explored?

It is important in a cohort study that a greater percentage of people are followed up. As a general guideline, at least 80% of patients should be followed up. Generally a dropout rate of 5% or less is considered insignificant. A rate of 20% or greater is considered to significantly impact on the validity of the study. However, in observational studies conducted over a lengthy period of time a higher dropout rate is to be expected. A decision on whether to include or exclude a study because of a high dropout rate is a matter of judgement based on the reasons why people dropped out, and whether dropout rates were comparable in the exposed and unexposed groups.

Reporting of efforts to follow up participants that dropped out may be regarded as an indicator of a well conducted study. Look for clear and justifiable description of why people were left out, excluded, dropped out etc. If there is no clear description or a statement in this regards, this will be a 'No'.

Q10. Were strategies to address incomplete follow up utilized?

Some people may withdraw due to change in employment or some may die; however, it is important that their outcomes are assessed. Selection bias may occur as a result of incomplete follow up. Therefore, participants with unequal follow up periods must be taken into account in the analysis, which should be adjusted to allow for differences in length of follow up periods. This is usually done by calculating rates which use person-years at risk, i.e. considering time in the denominator.

Q11. Was appropriate statistical analysis used?

As with any consideration of statistical analysis, consideration should be given to whether there was a more appropriate alternate statistical method that could have been used. The methods section of cohort studies should be detailed enough for reviewers to identify which analytical techniques were used (in particular, regression or stratification) and how specific confounders were measured.

For studies utilizing regression analysis, it is useful to identify if the study identified which variables were included and how they related to the outcome. If stratification was the analytical approach used, were the strata of analysis defined by the specified variables? Additionally, it is also important to assess the appropriateness of the analytical strategy in terms of the assumptions associated with the approach as differing methods of analysis are based on differing assumptions about the data and how it will respond.

**Reference**

<https://jbi.global/critical-appraisal-tools>

**Supplementary file 2. Critical appraisal of included studies**

| **Case-control study** | **Q1** | **Q2** | **Q3** | **Q4** | **Q5** | **Q6** | **Q7** | **Q8** | **Q9** | **Q10** | **Total** | **Risk of bias** |
| --- | --- | --- | --- | --- | --- | --- | --- | --- | --- | --- | --- | --- |
| Enzo Grossi et al., 2016 | Y | Y | Y | Y | Y | Y | U | Y | Y | Y | 90% | LOW |
| J.W. Miller et al., 2002 | Y | Y | Y | Y | Y | Y | Y | Y | Y | Y | 100% | LOW |
| Leon Veryard et al., 2013 | N | Y | Y | Y | Y | Y | Y | Y | Y | Y | 90% | LOW |
| Michael L. Selley et al., 2004 | Y | Y | Y | Y | Y | Y | Y | Y | Y | Y | 100% | LOW |
| Fabrizio Piazza et al., 2012 | N | Y | Y | Y | Y | Y | Y | Y | N | Y | 80% | LOW |
| Pierpaola Tannorella et al., 2015 | Y | Y | Y | Y | Y | Y | Y | Y | Y | Y | 100% | LOW |
| Carlo Cervellati et al., 2013 | Y | Y | Y | Y | Y | Y | Y | Y | Y | Y | 100% | LOW |
| Robert Clarke et al., 1998 | Y | Y | Y | Y | Y | Y | Y | Y | Y | Y | 100% | LOW |
| Małgorzata Bednarska-Makaruk et al., 2016 | Y | Y | Y | Y | Y | Y | Y | Y | Y | Y | 100% | LOW |
| ANDREW McCADDON et al., 1998 | Y | Y | Y | Y | Y | Y | Y | Y | Y | Y | 100% | LOW |

| **Cross-sectional study** | **Q1** | **Q2** | **Q3** | **Q4** | **Q5** | **Q6** | **Q7** | **Q8** | **Total** | **Risk of bias** |
| --- | --- | --- | --- | --- | --- | --- | --- | --- | --- | --- |
| Mustafa Cankurtaran et al., 2013 | Y | Y | Y | Y | Y | Y | Y | Y | 100% | LOW |
| B. Frick et al., 2006 | U | U | Y | Y | N | N | Y | Y | 50% | MODERATE |
| Grzegorz Raszewski et al., 2016 | U | U | Y | Y | N | N | Y | Y | 50% | MODERATE |
| Noel G. Faux et al., 2011 | Y | Y | Y | Y | Y | Y | Y | Y | 100% | LOW |
| Loïc Dayon et al., 2017 | Y | U | Y | Y | Y | Y | Y | Y | 80.75% | LOW |
| Robert Stewart et al., 2001 | Y | Y | Y | Y | Y | Y | Y | Y | 100% | LOW |
| Gaetano Corso et al., 2017 | Y | U | Y | Y | Y | Y | Y | Y | 80.75& | LOW |
| Sanja Devčić et al., 2014 | U | U | Y | Y | Y | Y | Y | Y | 75% | LOW |
| Martin Lehmann et al., 1999 | Y | Y | Y | Y | Y | Y | Y | Y | 100% | LOW |

| **Cohort study** | **Q1** | **Q2** | **Q3** | **Q4** | **Q5** | **Q6** | **Q7** | **Q8** | **Q9** | **Q10** | **Q11** | **Total** | **Risk of bias** |
| --- | --- | --- | --- | --- | --- | --- | --- | --- | --- | --- | --- | --- | --- |
| Babak Hooshmand et al., 2019 | U | Y | Y | Y | Y | Y | Y | Y | U | U | Y | 72.7% | LOW |
| Imrich Blasko et al., 2008 | Y | Y | Y | Y | Y | Y | Y | Y | Y | N | Y | 90.9% | LOW |
| Francisca A. de Leeuw et al., 2020 | Y | Y | Y | Y | Y | Y | Y | Y | Y | U | Y | 90.9% | LOW |
| Francisca A. de Leeuw et al., 2020 | Y | Y | Y | Y | Y | Y | Y | Y | N | N | Y | 81.8% | LOW |
| Sylvia Annerbo et al., 2009 | Y | Y | Y | Y | Y | Y | Y | Y | N | U | Y | 81.8% | LOW |
| SUDHA SESHADRI et al., 2002 | Y | Y | Y | Y | Y | Y | Y | Y | N | N | Y | 81.8% | LOW |
| Sylvia Annerbo et al., 2005 | Y | Y | Y | Y | U | Y | Y | Y | Y | Y | Y | 90.9% | LOW |
| Sylvia Annerbo et al., 2006 | Y | Y | Y | Y | Y | Y | Y | Y | Y | Y | Y | 100% | LOW |
| Andrew McCaddon et al., 2001 | Y | Y | Y | Y | Y | Y | Y | Y | Y | U | Y | 90.9% | LOW |
| M. BUDGE et al.,2000 | Y | Y | Y | Y | Y | Y | Y | Y | Y | Y | Y | 100% | LOW |

Source: Elaborated by the authors

Abbreviations: Y= Yes; N= No; U= Unclear; NA= Not/Applicable; Q= Questions.
