# Supplementary material for: Blood levels of circulating methionine components in Alzheimer’s disease and mild cognitive impairment: A systematic review and meta-analysis
Source: Front Aging Neurosci. 2022 Jul 22;14:934070. doi: 10.3389/fnagi.2022.934070 (PMC9354989; doi:10.3389/fnagi.2022.934070)
Supplement: Supplementary file 2 [file Data_Sheet_2.docx]

**Supplementary Material.** Search strategies

849 of PubMed

| Search number | Query | Results |
| --- | --- | --- |
| #1 | (biomarker*) OR (biological marker*) | 1,049,681 |
| #2 | (blood) OR (serum) OR (plasma) OR (RBC*) OR (erythrocyte*) OR (leukocyte*) OR (platelet*) OR (thrombocyte*) | 6,193,165 |
| #3 | (methionine) OR (Met) OR (S-adenosylmethionine) OR (S-adenosyl methionine) OR (S-adenosyl-L-methionine) OR (SAM) OR (SAMe) OR (AdoMet) OR (S-adenosyl homocysteine) OR (S-adenosyl-L-homocysteine) OR (SAH) OR (AdoHcy) OR (amino acid*) OR (methylation) OR (Transmethylation) OR (methyl group) OR (methyl donor) | 3,016,060 |
| #4 | (Alzheimer Disease [MeSH Terms]) OR (Alzheimer*) OR (cognitive impairment) | 285,201 |
| #5 | #1 AND #2 AND #3 AND #4 | 849 |

921 of Embase

| NO. | Query | Results |
| --- | --- | --- |
| #1 | Biomarker* OR 'biological marker*' | 487,975 |
| #2 | blood OR serum OR plasma OR rbc* OR erythrocyte* OR leukocyte* OR platelet* OR thrombocyte* | 7,274,296 |
| #3 | methionine OR met OR 's adenosylmethionine' OR 's-adenosyl methionine' OR 's adenosyl l methionine' OR sam OR same OR adomet OR 's-adenosyl homocysteine' OR 's adenosyl l homocysteine' OR sah OR adohcy OR 'amino acid*' OR methylation OR transmethylation OR 'methyl group' OR 'methyl donor' | 3,268,141 |
| #4 | 'alzheimer disease' OR alzheimer* OR 'cognitive impairment' | 363,876 |
| #5 | #1 AND #2 AND #3 AND #4 | 921 |

214 of The Cochrane Library

| ID | Search | Hits |
| --- | --- | --- |
| #1 | biomarker* OR biological marker* | 48,858 |
| #2 | blood OR serum OR plasma OR RBC* OR erythrocyte* OR leukocyte* OR platelet* OR thrombocyte* | 479,009 |
| #3 | methionine OR Met OR S-adenosylmethionine OR S-adenosyl methionine OR S-adenosyl-L-methionine OR SAM OR SAMe OR AdoMet S-adenosyl homocysteine OR S-adenosyl-L-homocysteine OR SAH OR AdoHcy OR amino acid* OR methylation OR Transmethylation OR methyl group OR methyl donor | 165,747 |
| #4 | Alzheimer Disease OR Alzheimer* OR cognitive impairment | 24,643 |
| #5 | #1 AND #2 AND #3 AND #4 | 214 |

2,287 of Web of science

| ID | Search | Results |
| --- | --- | --- |
| #1 | Biomarker (Topic) or biological marker∗(Topic) | [467,896](http://apps.webofknowledge.com/summary.do?product=UA&doc=1&qid=959&SID=6COXGwZSwWxP9dHFJu3&search_mode=GeneralSearch&update_back2search_link_param=yes) |
| #2 | Blood (Topic) or serum (Topic) or plasma (Topic) or RBC* (Topic) or erythrocyte* (Topic) or leukocyte* (Topic) or platelet* (Topic) or thrombocyte* (Topic) | 12,268,559 |
| #3 | Methionine (Topic) or Met (Topic) or S-adenosylmethionine (Topic) or S-adenosyl methionine (Topic) or S-adenosyl-L-methionine (Topic) or SAM (Topic) or AdoMet (Topic) or S-adenosyl homocysteine (Topic) or S-adenosyl-L-homocysteine (Topic) or SAH (Topic) or AdoHcy (Topic) or amino acid* (Topic) or methylation (Topic) or transmethylation (Topic) or methyl group (Topic) or methyl donor (Topic) | [10,292,743](http://apps.webofknowledge.com/summary.do?product=UA&doc=1&qid=964&SID=6COXGwZSwWxP9dHFJu3&search_mode=GeneralSearch&update_back2search_link_param=yes) |
| #4 | Alzheimer Disease (Topic) or Alzheimer* (Topic) or cognitive impairment (Topic) | [537,013](http://apps.webofknowledge.com/summary.do?product=UA&doc=1&qid=965&SID=6COXGwZSwWxP9dHFJu3&search_mode=GeneralSearch&update_back2search_link_param=yes) |
| #5 | #1 AND #2 AND #3 AND #4 | 2,287 |
