# Supplementary figures and images for: Blood levels of circulating methionine components in Alzheimer’s disease and mild cognitive impairment: A systematic review and meta-analysis
Source: Front Aging Neurosci. 2022 Jul 22;14:934070. doi: 10.3389/fnagi.2022.934070 (PMC9354989; doi:10.3389/fnagi.2022.934070)

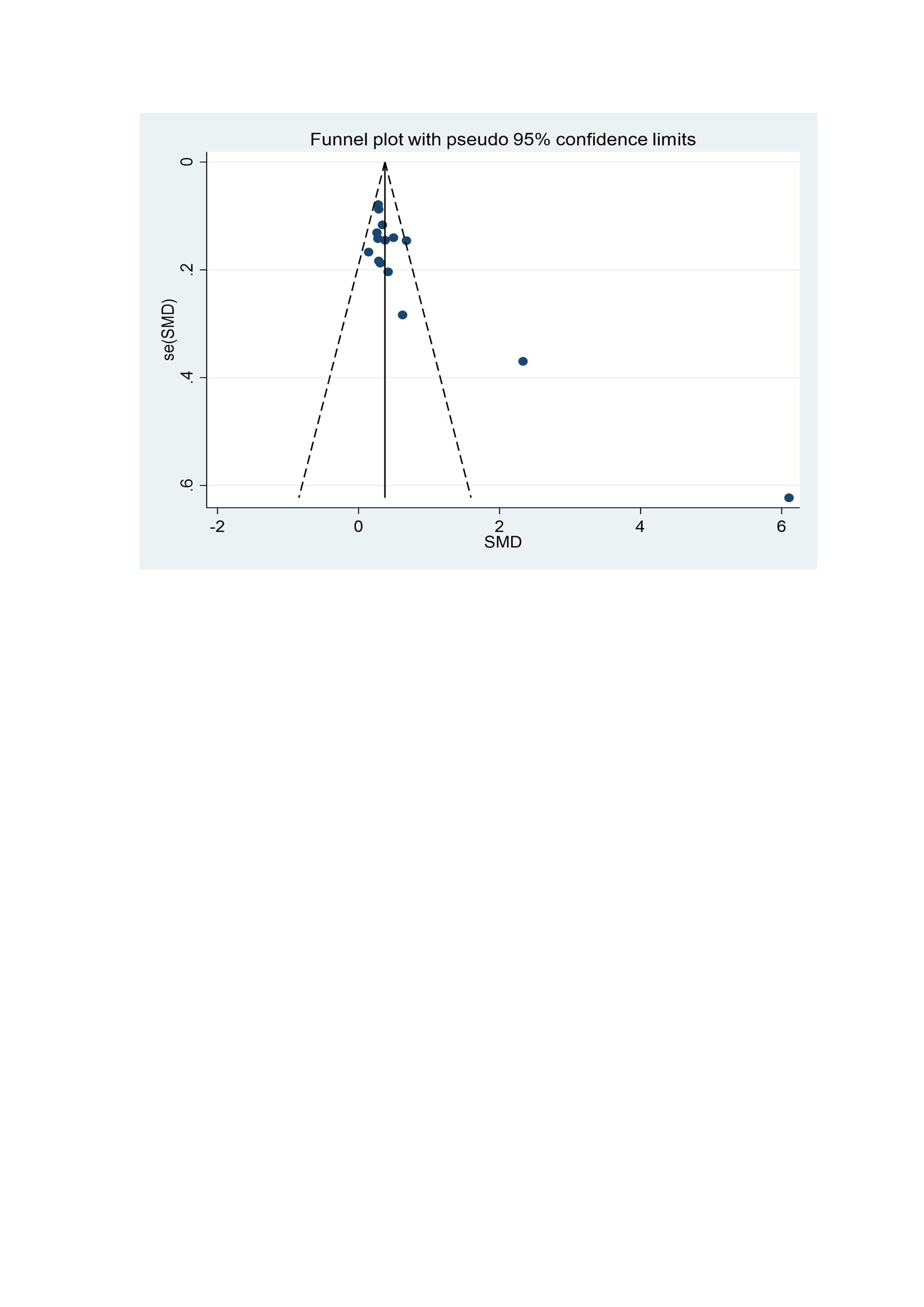

Supplement: Supplementary Figure 1 — Funnel plot with pseudo 95% confidence limits of 15 studies comparing blood Hcy levels between AD vs. Control. [file Image_1.JPEG]

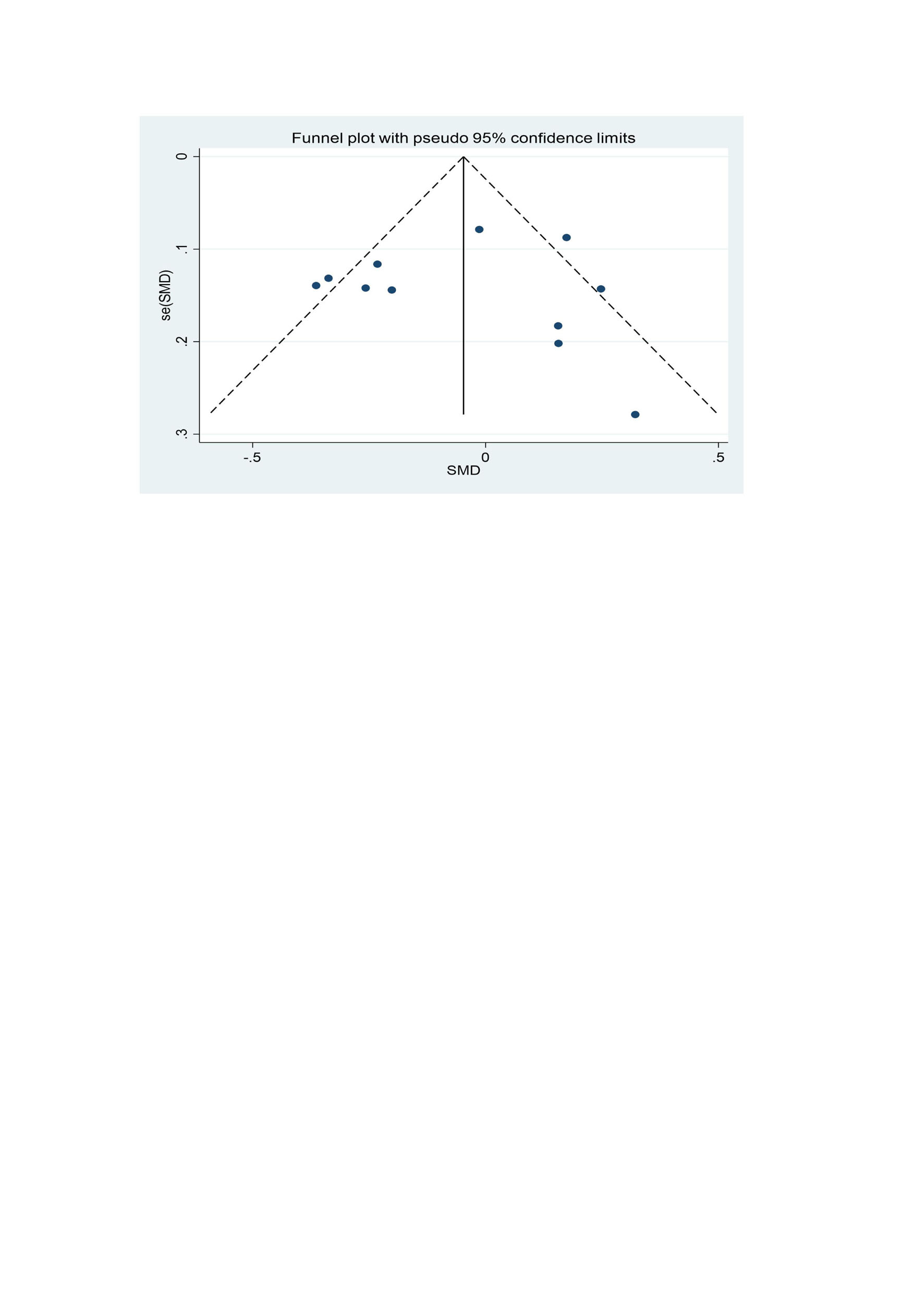

Supplement: Supplementary Figure 2 — Funnel plot with pseudo 95% confidence limits of 11 studies comparing blood vitamin B12 levels between AD vs. Control. [file Image_2.JPEG]
